# Nonequilibrium Thermodynamics of Polymeric Liquids via Atomistic Simulation

**DOI:** 10.3390/e24020175

**Published:** 2022-01-25

**Authors:** Brian Joseph Edwards, Mohammad Hadi Nafar Sefiddashti, Bamin Khomami

**Affiliations:** Materials Research and Innovation Laboratory, Department of Chemical and Biomolecular Engineering, University of Tennessee, Knoxville, TN 37996, USA; mnafarse@vols.utk.edu (M.H.N.S.); bkhomami@utk.edu (B.K.)

**Keywords:** polymeric liquids, nonequilibrium entropy, nonequilibrium molecular dynamics, elongational flow, shear flow

## Abstract

The challenge of calculating nonequilibrium entropy in polymeric liquids undergoing flow was addressed from the perspective of extending equilibrium thermodynamics to include internal variables that quantify the internal microstructure of chain-like macromolecules and then applying these principles to nonequilibrium conditions under the presumption of an evolution of quasie equilibrium states in which the requisite internal variables relax on different time scales. The nonequilibrium entropy can be determined at various levels of coarse-graining of the polymer chains by statistical expressions involving nonequilibrium distribution functions that depend on the type of flow and the flow strength. Using nonequilibrium molecular dynamics simulations of a linear, monodisperse, entangled C_1000_H_2002_ polyethylene melt, nonequilibrium entropy was calculated directly from the nonequilibrium distribution functions, as well as from their second moments, and also using the radial distribution function at various levels of coarse-graining of the constituent macromolecular chains. Surprisingly, all these different methods of calculating the nonequilibrium entropy provide consistent values under both planar Couette and planar elongational flows. Combining the nonequilibrium entropy with the internal energy allows determination of the Helmholtz free energy, which is used as a generating function of flow dynamics in nonequilibrium thermodynamic theory.

## 1. The Trouble with Entropy

Equilibrium and nonequilibrium thermodynamics of polymeric liquids pose a demanding intellectual and practical challenge for scientists and engineers. Unlike simple, isotropic liquids, the macromolecular configurational microstructure of long, chain-like polymers adds a deeper level of complexity to an already challenging problem. Frameworks of equilibrium and nonequilibrium thermodynamics of polymers have been proposed over the previous 75 years [1,2,3,4,5,6,7,8,9,10], but explicit proofs of their validity and usefulness have yet to be provided, principally because of the troublesome fact that experimental measurement of the entropy of a polymeric liquid is not currently possible. With the advent of realistic macromolecular simulations of melts and solutions over the past decade, the same statement has been true of calculating the entropy from an atomistic simulation of a polymeric liquid; i.e., it has not been possible. After all, even for dilute gases the calculation is quite complicated.

At the simplest level of description, the microstructure of a polymer fluid can be expressed in terms of appropriate “internal variables,” each representing some element of the inherent configurational state of the macromolecular liquid. Expressed as a function called “internal energy,” $U=U(S,V,C_{i})$, one has an extended state space where the internal coordinates, $C_{i}\equiv C_{1},\;C_{2},\;\ldots$, along with the entropy, *S*, and the volume, *V*, can vary over a wide range of time and length scales. The mechanics of thermodynamic theory can then be applied to express this internal energy function (or its inverse, $S=S(U,V,C_{i})$) in terms of conjugate variables, such as pressure, *p*, and temperature, *T*. This allows reexpression of the internal energy function in terms of other variable sets via Legendre transformations without loss of thermodynamic information, leading to functions such as enthalpy, Helmholtz free energy, and Gibbs free energy [8]. Using standard to advanced experimental instruments and procedures of all sorts, most of these variables, such as *p*, *T*, and *V*, can be directly measured; even some microstructural variables $C_{i}$ can be routinely determined from experiments. Furthermore, all these quantities can be calculated directly via atomistic simulation. Although it is beyond the capacity of an experiment to determine the internal energy directly for polymeric liquids, it is possible to calculate it via molecular dynamics simulation where one begins with a classical potential model for the force interactions between neighboring atoms. (Of course, the accuracy of this simulation depends critically on the quality of the potential model.) What cannot be determined, however, either via experiment or simulation, is the liquid’s inherent entropy. There is no available experimental instrument or technique to measure, directly or indirectly, a unique value of the entropy of a polymeric liquid at a particular state point. Likewise, there exists no method for calculating the entropy of a polymeric fluid from atomistic simulation, and without the entropy, a critical piece of information is missing such that the powerful framework of equilibrium and nonequilibrium thermodynamics, while philosophically and aesthetically pleasing, cannot be applied to its full potential.

The authors do not know how to measure directly the entropy of a polymeric liquid via experiment nor do they even know where to begin searching for such a method. Therefore, herein they ignore this noble challenge and focus on calculating entropy (or at least changes in entropy) directly via realistic atomistic simulations, which would seem to be a much simpler matter to investigate. The problem with calculating the entropy, either from theory or simulation, is that its value changes depending on how one observes the system. This statement would seem absurd on that face of it, but it is true.

Consider a monodisperse polyethylene (PE) liquid composed of linear macromolecules, which is well described as a system of Gaussian chains, each chain composed of a certain number of Kuhn segments of about 16 Å in length [11,12,13] (about 12 successive methylene units). The characteristic topology of the ensemble of chains comprising the liquid is solely determined by a random walk from one Kuhn segment to the next over the entire length of the macromolecule, in which the internal configurational dynamics of each Kuhn segment are assumed to remain equilibrated on the time scale of interest to the system. Within the observation window of the experimenter (or simulator), which is generally on the order of the system’s longest time scale, the entropy can be calculated without regard to the internal dynamics of these equilibrated Kuhn segments, wherein the atomic bond lengths and bond angles remain practically unchanged by the configurational diffusion of the chain segments. Accordingly, the configurational state of the macromolecules can be determined solely by statistical analysis of the collection of Kuhn segments without regard to the internal dynamics of the segments. Upon application of flow, however, particularly high strain-rate flow, the observation window of the entropy expands toward shorter time scales that were not activated before. At high enough strain rates, the bond lengths, bond angles, and dihedral angles are activated, and the internal dynamics of the Kuhn segments play a large role in the system’s response as the segments themselves are stretched and bent by the applied flow field. A calculation of the entropy at such a high strain rate must incorporate many more variations in configurational probabilities.

The classical definition of entropy is

(1) $$S=-\;k_{B}\;\sum_{i}\;p_{i}\;\ln p_{i}=k_{B}\;\ln\;\Omega \;\;,$$

which is a logarithmic measure of the number of configurational states with a distinct probability of being occupied ($p_{i}$), and $k_{B}$ is Boltzmann’s constant. If all configurational states are equally probable, one has the second equality where $p_{i}=1/\Omega$, with Ω representing the number of distinct configurational microstates. Under quiescent conditions, when the observation window of the experimenter is normally only engaged in long time scales, the calculation of the entropy should yield the same value whether or not the internal Kuhn dynamics are counted. This is because the internal Kuhn segment configurations are not changing on average and hence not affecting the overall configurational probability distribution of the entire system. However, at high strain rates where the observation window has been extended to small time scales and the internal dynamics of the Kuhn segments have been activated, calculation of the entropy depends upon which observation window is used during the computation. Indeed, how the system is observed even affects the calculation of the internal energy at high strain rates. Within the system with the smaller observation window (i.e., the one restricted to long time scales), the macromolecular chains composed of Kuhn steps can be considered as freely jointed chains without energetic interactions between the segments, such that the configurational state of the system can be determined in a purely statistical fashion. This implies and requires no change in the internal energy state of the fluid as a function of the strain rate. However, within the observation window extended to small time scales, the internal configurational changes within the Kuhn segments directly impact the interatomic potential energy and resultant forces between neighboring atoms, thus producing a definite change in the internal energy as a function of the strain rate. The question then arises, in order to calculate the Helmholtz free energy of the fluid according to the Legendre transformation A=U−TS, which is the appropriate observation window to use? Clearly, the choice should affect the resulting value of *A*.

One might expect that the most accurate value of entropy, and also internal energy, would be obtained by performing the calculation over the largest observation window possible, thereby incorporating all possible configurational states of the macromolecular system at all length scales and time scales, regardless of how large or small these scales might be; this expectation is not unreasonable but not necessarily always practicable. A possibly better method to approach the problem is to begin by considering the implications of thermodynamics of systems with internal variables.

## 2. Thermodynamics with Internal Variables

A key assumption in equilibrium thermodynamics is that the time scale for changes in the system is sufficiently large as compared to the time scales of any relevant internal variables within the system [8]. Consequently, it can be assumed that the internal variables are always occupying equilibrium microstates corresponding to the state space of the large time-scale thermodynamic variables of the system, such as *V* and *S*. When the system depends on internal variables with time scales on the order of the time scales for physical changes within the system, it still might be possible to describe the system sufficiently well through an extended equilibrium relation, provided that the thermodynamic state space is enlarged to include internal variables accounting for intermediate microstructural states. In this scenario of a hierarchy of system time scales, irreversibility is invariably introduced into the system as the internal variables progress toward their equilibrium microstates within shorter time increments than the one characterizing the overall system response time. Hence, an appropriate definition of the observation window, i.e., the range of time and length scales over which the important thermodynamic variables are defined, is crucial to a proper physical description of the thermodynamic properties of a system approaching equilibrium. Variables that relax over time scales much larger than the window of observation are effectively “frozen” at specific values, essentially acting merely as parameters of the dynamic system response. On the other hand, variables with time scales much smaller than the observation window are “equilibrated” at values that are determined by the instantaneous values of the “dynamic” variables that are activated within the relevant observation window.

In the example of the PE liquid of Section 1, within an observation window on the order of a picosecond, the vibrations of the bond lengths and bond angles (and occasional transitions of the dihedral angles) comprise the state space of thermodynamic internal variables, whereas the larger time-scale Kuhn segments are effectively immobile (i.e., frozen). However, when the observation window is moved to larger times, on the order of nanoseconds, the bond-length and bond-angle oscillations are essentially equilibrated in their equilibrium microstates and do not affect the motions of the Kuhn segments. During a nonequilibrium process, however, such as a strong imposed flow field, all relaxation modes of the system are activated and must be accounted for within an appropriately scaled observation window. As the flow rate increases, the observation window widens to stimulate previously equilibrated modes. Upon cessation of flow, the short time-scale modes rapidly re-equilibrate into microstates consistent with the instantaneous states of the long time-scale modes (which appear frozen to the short time scale modes). On the other end of the time spectrum, the long time-scale modes slowly relax back to their equilibrium condition prior to the application of flow with the short time-scale modes instantaneously re-equilibrating at each step along the way as the slow overall relaxation process progresses. This expansion of the appropriate observation window under nonequilibrium conditions ultimately results in the complexity of calculating entropy under external perturbations, as illustrated below.

Consider a closed thermodynamic system (i.e., constant mass) comprised of a single internal variable, *X*, such that the state space of system can be duly extended to express the internal energy as U(S,V,X). A conjugate variable to *X* is defined as x≡∂U/∂X, such that changes in the internal energy can be expressed as

(2) dU=TdS−pdV+xdX.

Let τ be the time scale associated with the dynamic variables *S* and *V*, and let $\tau_{X}$ be the time scale of the internal variable *X*. Assuming $\tau_{X}\ll \tau$, a system perturbed from its equilibrium condition by an amount dP will initially experience a fast relaxation of the internal variable *X* such that xdX≤0, with the equality valid once “internal equilibrium” is attained, at which point xdX=0 and $X^{eq}\left(S,V\right)$ is the equilibrated internal variable whose instantaneous value is determined by the current values of the dynamic variables, *V* and *S*.

Describing the system in terms of the entropy function, S(U,V,X), inequality xdX≤0 implies that

(3) TdS(U,V,X)=dU+pdV−xdX≥TdS(U,V)=dU+pdV,

with the equality valid only after internal equilibrium has been attained. This expression indicates that the variation in entropy is greater in the extended state space since dS(U,V,X)≥dS(U,V), which implies the opposite inequality for the entropy function,

(4) S(U,V,X)≤S(U,V).

This inequality can be extended to include additional internal variables (Y,Z,…) relaxing over ever-decreasing time scales $\tau_{Z}\ll \tau_{Y}\ll \tau_{X}\ll \tau$:

(5) …≤S(U,V,X,Y,Z)≤S(U,V,X,Y)≤S(U,V,X)≤S(U,V).

**Figure 1:**
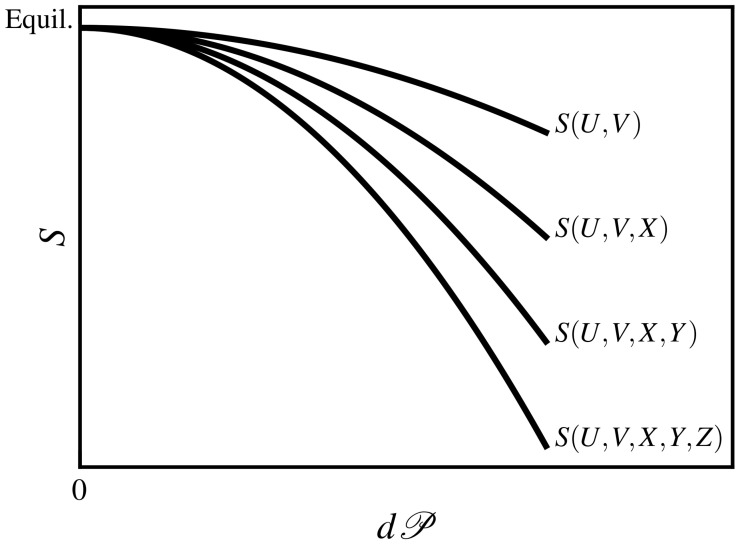
Entropy expressed as functions of extended state space upon application of a perturbation (dP) from equilibrium. Extending the state space decreases the entropy away from equilibrium, but in all cases a universal maximum in entropy is attained as equilibrium is restored.

This expression indicates that as more variables attain internal equilibrium, and are thus no longer necessary for the thermodynamic description of the system, the entropy monotonically increases. Hence, a more-detailed description of state implies an entropy of smaller magnitude. This concept is illustrated in Figure 1. Note that entropy is a maximum at equilibrium, where all state spaces provide an equivalent value; however, away from equilibrium, the choice of the observation window can significantly impact the calculation of the entropy in possibly unanticipated ways.

## 3. Nonequilibrium Thermodynamics of Polymeric Liquids and the Entropy Calculation

A solid framework of nonequilibrium thermodynamics of polymeric fluids under an external flow field was expounded by Beris and Edwards [8] in terms of an extended thermodynamic state space composed of internal variables representing the fluid’s microstructure over a range of time and length scales. An extended Gibbs relation was expressed in terms of the internal energy function, $U=U(S,V,C_{i})$, where internal variables $C_{i}\equiv C_{1},\;C_{2},\;\ldots$ represented various measures of the internal configurational space of the flowing medium. A single microstructural variable (for simplicity) is now assumed to be the second-order dimensionless conformation (second moment) tensor,

(6) $$C\equiv \frac{3}{R_{eq}^{2}}\;\int \;RR\;\psi \left(R\right)\;d^{3}R=\frac{\langle 3\;RR\rangle }{R_{eq}^{2}}\;\;,$$

where R is the end-to-end vector of a specific polymer macromolecule, $R_{eq}^{2}$ is the mean-square magnitude of that vector in the unperturbed state, and ψ(R) is its instantaneous probability distribution function. The extended Gibbs equation is then [8]

(7) $$dU=TdS-p\;dV+Z_{\alpha \beta }\;dC_{\alpha \beta }\;\;,$$

where Z can be viewed as an intensive thermodynamic conjugate variable (analogously to *T* and *p*) associated with the configurational potential of the nonequilibrium microstructural state represented by C. (Note that the Einstein summation convention over repeated indices was employed.) In essence, Z can be associated with the magnitude of an imposed external deformation just as its association with the derivative of an energy function through the Principle of Virtual Work (of a deformable body) might suggest. Hence, it can be viewed as a thermodynamic force derived from an applied deformation. Because *U* is a thermodynamic state variable and exact differential,

(8) $$dU=\frac{\partial U}{\partial S}\;dS+\frac{\partial U}{\partial V}\;dV+\frac{\partial U}{\partial C_{\alpha \beta }}\;dC_{\alpha \beta }\;\;,$$

which implies that functional relationships exist between the internal energy and the thermodynamic conjugate variables,

(9) $$\frac{\partial U}{\partial S}=T\;\;,\;\;\;\;\frac{\partial U}{\partial V}=-p\;\;,\;\;\;\;\frac{\partial U}{\partial C_{\alpha \beta }}=Z_{\alpha \beta }\;\;.$$

The equality of mixed partial derivatives then requires Maxwell relationships between these variables, including those for the components of the conformation tensor [8],

(10) $$\frac{\partial T}{\partial V}=-\frac{\partial p}{\partial S}\;\;,\;\;\;\;\frac{\partial T}{\partial C_{\alpha \beta }}=\frac{\partial Z_{\alpha \beta }}{\partial S}\;\;,-\frac{\partial p}{\partial C_{\alpha \beta }}=\frac{\partial Z_{\alpha \beta }}{\partial V}\;\;,\;\;\;\frac{\partial Z_{\alpha \beta }}{\partial C_{\gamma \epsilon }}=\frac{\partial Z_{\gamma \epsilon }}{\partial C_{\alpha \beta }}\;\;.$$

Manipulating the internal energy function via a series of Legendre transformations allows for an equivalent set of thermodynamic information to be formulated in terms of other combinations of variables, resulting in the enthalpy, Helmholtz free energy, and Gibbs free energy, respectively:

(11) H(S,V,C)=U+pV,

(12) A(T,V,C)=U−TS,

(13) G(T,p,C)=H−TS=U+pV−TS.

Analogous expressions can also be obtained via applying the Legendre transformation to the conjugate variable Z as well,

(14) $$\tilde{U}\left(S,V,Z\right)=U-Z_{\alpha \beta }C_{\alpha \beta }\;\;,$$

(15) $$\tilde{H}\left(S,p,Z\right)=\tilde{U}+pV=U+pV-Z_{\alpha \beta }C_{\alpha \beta }\;\;,$$

(16) $$\tilde{A}\left(T,V,Z\right)=\tilde{U}-TS=U-TS-Z_{\alpha \beta }C_{\alpha \beta }\;\;,$$

(17) $$\tilde{G}\left(T,p,Z\right)=\tilde{H}-TS=U+pV-TS-Z_{\alpha \beta }C_{\alpha \beta }\;\;.$$

which are expressed in terms of some applied external deformation of the microstructure of the polymeric liquid; such a construct has been employed before [8,14,15]. It must be recognized, however, that these new functions are not equivalent to the original internal energy, enthaply, Helmholtz energy, and Gibbs energy. In this framework, the conformation tensor is the thermodynamic conjugate variable to the applied deformation, $C=-\partial \tilde{U}/\partial Z$, which can be recognized by taking the differential of Equation (14) and applying Equation (7), $d\tilde{U}=TdS-pdV-C_{\alpha \beta }dZ_{\alpha \beta }$, where $dZ_{\alpha \beta }$ can be viewed as an externally applied deformation. Taking exact differentials of energy functions (14)–(17) then yields Maxwell relationships involving the conjugate variable Z, which are similar to those of Equation (10).

The above expressions make possible the calculation of thermodynamic quantities relevant to virtually any ensemble, even those at constant deformation potential, Z. However, all of this theoretical machinery is impeded by the necessity of appropriately evaluating the entropy. In principle, one might expect that calculating the entropy would not present such a significant problem, since it is primarily a purely statistical computation, but in practice this calculation depends on the number and nature of the variables used to represent the configurational state of the liquid, as discussed in Section 2.

In the example of the linear PE liquid at equilibrium, the chain-like molecules each have an enormous number of possible configurational states that they can inhabit. When a PE molecule is fully extended with all bond lengths, bond angles, and dihedral angles occupying *trans* conformations, it lies completely within a single plane, and there is only one distinct configuration that is consistent with this chain conformation. However, at equilibrium, the polymer macromolecules assume random coils of a certain dimension rather than extending appreciably, which is because there are innumerably more configurations that are consistent with this random coil. Although each configuration is equally probable to occur as the fully extended one ($p_{i}=1/\Omega$), the mere fact that there is such an enormous number of possible distinct configurational states mandates that the individual molecules assume random coils of dimensions commensurate with a Gaussian distribution around the mean. So, in the absence of any interatomic forces that might slightly distort this distribution, the chain conformations are entirely determined by statistical considerations. Under application of an external flow field, this equilibrium distribution is distorted in a way that is difficult to determine *a priori*, and this is where atomistic simulations can play a prominent role in the proper determination of the system entropy. However, even such a detailed simulation allows multiple, thermodynamically valid ways to perform the entropy calculation.

### 3.1. Calculation of Entropy at the Mesoscale

Consider the configurational state of a linear, monodisperse PE melt. The most probable configurational state is the one occupied under quiescent conditions at a given temperature and volume/pressure. The entropy of this state can be denoted as $S_{eq}$, and it can be calculated in principle by Equation (1) once the probabilities $p_{i}$ are known. Away from equilibrium, the entropy of the system will be lower than the equilibrium value if the flow induces a configurational state upon the melt that is not consistent with the most probable one. This entropy decrease can be quantified via Equation (1) by taking the natural logarithm of the ratio of the number of distinct configurations compatible with the respective microstructural states,

(18) $$\Delta S=S-S_{eq}=k_{B}\;\ln\left(\frac{\Omega }{\Omega_{eq}}\right)\;\;.$$

Equivalently, the entropy difference can be expressed in terms of the joint probabilities of finding the liquid in the respective configurational microstates,

(19) $$\Delta S=k_{B}\;\ln\left(\frac{P}{P_{eq}}\right)\;\;.$$

Let a certain number of macromolecular chains (the following analysis applies not only to the full chain whose configurations are expressed in terms of the end-to-end vector, R, but also to internal chain segments of arbitrary length), $n_{i}$, occupy configurations in which the variable R assumes values between $R_{i}$ and $R_{i}+dR_{i}$, with $p_{i}$ being the probability that a given molecule adopts this particular configuration. Then, the joint probability of finding the system occupying the configurational state represented by $n_{1},n_{2},n_{3},\ldots$ is expressed by [16]

(20) $$P=N!\;\prod_{i}\;\frac{p_{i}^{n_{i}}}{n_{i}!}\;\;,$$

where *N* is the total number of molecules. Note that $n_{i}=Np_{i}$ and that the end-to-end vector, R, was used as the discretization variable representing the configurational microstate of the melt; however, this choice is arbitrary and can be adjusted appropriately as the physical circumstances warrant, as explained below. The ratio of the nonequilibrium to equilibrium joint probabilities is then

(21) $$\frac{P}{P_{eq}}=\prod_{i}\;\frac{n_{eq,i}!}{n_{i}!}\;p_{i}^{\left(n_{i}-n_{eq,i}\right)}\;\;,$$

and after introducing Stirling’s approximation in the form $n!\approx {\left(n/e\right)}^{n}$, this ratio, as derived by F.T. Wall [16], is

(22) $$\begin{array}{c} \frac{P}{P_{eq}}=\prod_{i}\;\frac{{\left(\frac{n_{eq,i}}{e}\right)}^{n_{eq,i}}}{{\left(\frac{n_{i}}{e}\right)}^{n_{i}}}\;p_{i}^{\left(n_{i}-n_{eq,i}\right)}=\prod_{i}\;\frac{{\left(\frac{n_{eq,i}}{e}\right)}^{n_{eq,i}}{\left(\frac{n_{eq,i}}{e}\right)}^{n_{i}}{\left(\frac{n_{eq,i}}{e}\right)}^{-n_{i}}}{{\left(\frac{n_{i}}{e}\right)}^{n_{i}}}\;p_{i}^{\left(n_{i}-n_{eq,i}\right)}\\ =\prod_{i}\;{\left(\frac{n_{eq,i}}{n_{i}}\right)}^{n_{i}}{\left(\frac{n_{eq,i}}{ep_{i}}\right)}^{\left(n_{eq,i}-n_{i}\right)}=\prod_{i}\;{\left(\frac{n_{eq,i}}{n_{i}}\right)}^{n_{i}}\;\;, \end{array}$$

or in logarithmic form as

(23) $$\ln\left(\frac{P}{P_{eq}}\right)=\sum_{i}\;n_{i}\;\ln\left(\frac{n_{eq,i}}{n_{i}}\right)\;\;.$$

Combining Equations (19) and (23) provides Wall’s expression for the entropy change,

(24) $$\Delta S=k_{B}\sum_{i}\;n_{i}\;\ln\left(\frac{n_{eq,i}}{n_{i}}\right)\;\;.$$

Wall’s equation can also be expressed in terms of the probability distributions associated with the various configurational states represented by the $R_{i}$ as $n_{eq,i}=N\psi_{eq}\left(R_{i}\right)d^{3}R_{i}$ and $n_{i}=N\psi \left(R_{i}\right)d^{3}R_{i}$ as

(25) $$\Delta S=Nk_{B}\int \;\psi \;\ln\left(\frac{\psi_{eq}}{\psi }\right)\;d^{3}R\;\;.$$

The Wall expression allows calculation of the entropy change under an applied external flow field from direct knowledge of the equilibrium and nonequilibrium distribution functions of the end-to-end vector, which, at least in principle, can be obtained from an atomistic NEMD simulation. Furthermore, there is nothing physically restrictive of the above analysis to the end-to-end vector, and any arbitrary vector spanning a subsection of the linear chain-like molecule would provide an equally valid descriptor of the configurational microstate of the system, albeit on a possibly very different length and time scale as compared to R: when the chain subsection is successively reduced in length, the time scale upon which the chain segment is activated is diminished. Indeed, there is also no reason to restrict the system description to only a single internal variable, and multiple vectors representing various subsections of the chain can also be used for the determination of the system entropy, as in the Rouse Model [17] discussed below.

A model for describing the multimode dynamics of a dilute polymer solution was developed by P. E. Rouse in the early 1950s by representing a long-chain macromolecule as a series of n+1 beads connected by *n* Gaussian springs. (Probably not coincidentally, Rouse was a Ph.D. student of Wall’s in the early 1940s at the University of Illinois.) In this mesoscopic model, subsequently known as the Rouse Model and earning Rouse the 1966 Bingham Medal of the Society of Rheology, a polymer chain is represented as a series of connected vectors spanning subsections of the entire chain, each section representing at least enough Kuhn segments such that the distribution of the random segmental configurations from one end of the chain subsection to the other end is Gaussian around the mean. Hence, the configuration of the entire chain consisting of *n* submolecules is quantified by the corresponding set of *n* end-to-end vectors spanning each submolecule. Expression of this system of vectors in terms of normal component vectors revealed that the dynamics of the polymer solution could be conceptualized as a translational motion of the molecular center of mass (mode k=0) and a spectrum of internal relaxation modes corresponding to independent dynamics of successively shorter chain portions by iterative elimination of submolecules. The time scales ($\tau_{k}$) associated with these *k* (1≤k≤n) relaxation modes are successively smaller as the mode number becomes larger (or as the chain becomes shorter), diminishing proportionally as $1/k^{2}$. The longest of these internal relaxation times is commonly referred to as the Rouse time of the polymeric liquid, $\tau_{1}\equiv \tau_{R}$.

Booij applied Wall’s equation for the entropy change, Equation (25), to the probability distribution of the Rouse model expressed in terms of the second moments of the Gaussian-mode distributions [17,18],

(26) $$\psi \left(\rho_{1},\rho_{2},\ldots ,\rho_{n}\right)=\prod_{k=1}^{n}\;\psi_{k}\left(\rho_{k}\right)=\frac{1}{{\left(2\pi \right)}^{3n/2}}\;\prod_{k}\;\frac{1}{|\det\;C_{k}{|}^{1/2}}\;\exp\left(-\frac{1}{2}\rho_{k}^{T}\cdot C_{k}^{-1}\cdot \rho_{k}\right)\;\;,$$

as also expressed in normal coordinate vectors, $\rho_{k}$, which represent independent modes of motion for the statistical segments of the overall chain. This independence implies that higher (faster) modes effectively represent chains of fewer segments. The second moments corresponding to each normal mode, $C_{k}$, satisfy independent (with respect to mode number) linear ordinary differential equations of the upper-convected Maxwell form,

(27) $$\tau_{k}\left(\frac{DC_{k}}{Dt}-C_{k}\cdot \nabla v-\nabla v^{T}\cdot C_{k}\right)=C_{k}-\delta \;\;,$$

where ∇v is the velocity gradient tensor; δ is the Kronecker delta tensor; and D(·)/Dt denotes the material derivative. Booij derived an entropy expression in terms of the second moments of the normal-coordinate distribution functions of the Rouse Model by assuming the normal-mode Gaussian distributions of Equation (26) [18]:

(28) $$\Delta S=-\frac{Nk_{B}}{2}\left(\sum_{k}\mathrm{tr}\left(C_{k}-\delta \right)-\sum_{k}\ln|\det\;C_{k}|\right)\;\;.$$

The reader should recall the general limitations of the Rouse model; although they will not be discussed in detail here, notable is the assumption of Gaussian normal-mode distributions of nonequilibrium microstates associated with various subsections of the overall polymer macromolecule under an imposed flow. This assumption is generally valid only within the linear viscoelastic regime, which occurs at low flow strength; indeed, Equation (27) can describe quite well the behavior of dilute polymer solutions and unentangled melts within the linear viscoelastic flow region [8,19,20]. One might then expect that for entangled melts at high flow strength, where the nonequilibrium configurational distributions can hardly be considered Gaussian, the entropy expression of Equation (28) could not be used to quantify adequately the configurational entropy change induced under flow. As such, direct knowledge of the nonequilibrium distribution under an applied flow field is required so that the general expression of Equation (25) can be used to calculate the entropy. Note, however, that this expression is based on the end-to-end vector of the entire polymer molecule, and it is entirely reasonable to quantify the chain dynamics using a hierarchy of segmental end-to-end vectors spanning subsections of the whole chain at various length scales, as in the Rouse Model. For example, for an entangled linear PE melt, one might choose the overall chain end-to-end vector to quantify the slow relaxational process of a highly stretched melt returning to its quiescent condition over a time scale on the order of milliseconds or longer; i.e., on the order of its disengagement time, $\tau_{d}$. As a second variable, the overall chain can be subdivided into entanglement strands, composed of segments of length scale associated with the average chain distance between successive entanglements, with end-to-end vectors defined spanning the distance from one hypothetical entanglement point to another. This variable would likely relax on a time scale commensurate with the liquid’s Rouse relaxation time, $\tau_{R}$, which according to reptation theory is given by $\tau_{R}=\tau_{d}/3Z$, where *Z* is the average number of entanglements per molecule [21]. Consequently, $\tau_{R}$ can be several orders of magnitude smaller than the disengagement time. At a still smaller length scale, the overall chain can be subdivided into its Kuhn segments (about 16 Å based on equilibrium molecular dimensions) [11,12,13,22] and an end-to-end vector defined such that it spans the length of the segment. Such a variable would likely be activated only under strong flow, and its relaxation back to its quiescent configurational state would occur on a time scale of a few nanoseconds. Going a step further, the Kuhn segment could also be subdivided into two or more subsections spanned by vectors that account for the stretching and vibrations of individual bonds and bond angles, as well as fluctuations of the dihedral angles. The time scale for this variable is on the order of a few picoseconds. As the subsection length scale is successively reduced, the relevant variable is activated at a higher flow strength and re-equilibrated over a smaller time scale.

Ideally, one would choose several subsections of sufficient length, possibly as just described, to span the entire range of time scales that are activated under strong flows. In principle, all of the probability distributions expressed in terms of the relevant variable can be computed using NEMD simulations and inserted into Equation (25) to calculate the liquid entropy. Note, however, that the value of the entropy change calculated in this fashion is dependent upon the chosen variable set and the magnitude of the imposed flow, as discussed in Section 2 and depicted graphically in Figure 1. An important question to answer is, if the liquid’s configurational state is described in terms of more than one variable and its corresponding probability distribution, is the total entropy change calculated simply as an additive sum of the contributions from each distribution based on Equation (25)? That would seem like a reasonable suggestion, and the entropy has a long history of being taken as an additive property [8] but only provided that the chosen variables are effectively independent of each other in a dynamical sense. Such an independence can be intimated, but probably not assured, as long as the associated time scales of the chosen variables are of sufficiently different orders of magnitude. Examining the issue from another perspective, however, points to a different conclusion. If the entropy is calculated for some nonequilibrium stationary state (as induced by a strong flow field) according to Equation (25) for multiple variables with widely varying time scales and then the individual values summed up to obtain the total entropy, then might the possibility of some form of over counting of distinct configurational microstates exist? Such over counting is definitely plausible. One might view the entropy calculated at the shortest length scale to be the most accurate and comprehensive, since longer length-scale phenomena are invariably composed of the cumulative effects of the shorter length-scale phenomena. Therefore, from this perspective, the total entropy is simply that calculated using the variables at the shortest length and time scales as averaged over *all* relevant length and time scales.

In the Booij calculation of entropy using the Rouse Model, the overall probability distribution function based on a large number of normal modes, each representing smaller length and time scales than the preceding mode, is decomposed into a joint probability in terms of a distribution function expressing the microstructural statistics of each (successively smaller portion of the overall molecule) normal mode. According to Equation (28), the Booij total entropy is calculated as a sum of the entropy associated with each mode based on the second moments of the corresponding normal mode distributions. Theoretically, these second moments can be calculated for any imposed flow field via Equation (27), but these evolution equations for the second moments are only valid within the linear viscoelastic flow regime. Although these equations are independent for each normal mode, there can be a large number of them, and the primary issue is that they are not clearly separated with respect to the intrinsic time scales of the individual modes, $\tau_{k}$. These time scales vary as [17]$\tau_{k}\propto 1/k^{2}$. For a chain composed of 10 beads, the time scales vary over a range of two orders of magnitude, with 75% of the upper end of this range spanned by the longest relaxation time. The remaining eight modes span a single order of magnitude. For longer chains, the spanned range increases in terms of orders of magnitude, but still the vast majority of modes span only a single order. Hence, for such a large number of successively smaller time scales, the application of the thermodynamics of internal variables is questionable. This does not seem to be a reasonable way to calculate a thermodynamically meaningful value of entropy.

From a computational standpoint, calculating the entropy based on a spectrum of relaxation times borders on unmanageable. A much better way, in principle and in practice, would seem to be to base the calculation of entropy on a fewer (and manageable) number of modes with a clear separation of time scales, even if it were only approximate. Hence the hierarchy of internal variables as discussed in Section 2 is certainly relevant to the study of polymer dynamics. Obviously, a reasonable selection of the set of internal variables used to describe a particular polymer flow application should be made *a priori*. For example, if one is primarily interested in slow flows within the linear viscoelastic flow regime, one would only include the longer time-scale variables, which are activated under such conditions. Variables that are active at much smaller time scales are effectively always equilibrated and consequently would not contribute to the entropy calculation anyway. As the flow strength increases, however, the range of relevant time scales extends to smaller times, effectively expanding the time range of the observation window downward.

Once a proper set of internal variables has been chosen to represent the independent dynamical modes of the polymeric liquid, NEMD simulations can generate the corresponding probability frequency distributions that are necessary to calculate the entropy according to Equation (25); however, such distributions are subject to statistical uncertainties that are exacerbated by the small system sizes required to remain within the operating range of the available computational resources. Consequently, these distributions are only rough approximations of the true distributions and are subject to large errors in occupation frequencies of the small bins used to discretize the distributions with respect to the corresponding variables. As a further problem, the extreme ends of these distributions are cut off, with bins at the fringes being completely unoccupied over the course of a simulation, which causes null values of the distributions at finite values of the variables. These null values can cause significant errors when calculating the entropy via logarithmic functions, such as the one appearing in Equation (25). Hence this equation, albeit theoretically exact for the chosen variable or variable set, is very difficult to implement in practice.

Given the computational difficulty in producing sufficiently smooth probability distributions of the chosen variables, another approach would be to calculate the entropy in terms of suitable averages of these distributions, such as provided by the second moments of the distribution that are calculated analogously to Equation (6). Indeed, this is exactly the concept followed by Booij in obtaining an expression for the entropy in terms of the second moments of the Rouse Model, except that he restricted the distributions to those obeying the Gaussian statistics of subdivided chain modes. Unfortunately, a similar expression in terms of second moments derived from a general (non-Gaussian) distribution is not available, and these distributions are typically highly non-Gaussian for polymer solutions and melts outside of the linear viscoelastic flow regime. However, if the second moments are computed directly from the simulation, in accordance with Equation (6), then they have effectively accounted for, at least in the mean, the non-Gaussian character of the relevant distribution function under flow. Upon insertion of these second moments into Equation (28), one effectively has some kind of quantifiable measure of the approximate entropy change in the system. This amounts to the assumption that Equation (28) remains at least approximately valid even if the second moments are not calculated based on Gaussian normal-mode distributions, as derived by Booij, but are instead computed directly from the NEMD simulation analogously to Equation (6). Hence, whatever nonequilibrium distributions are produced by the simulated system are appropriately quantified in the calculation of the second moments, and assuming that the nature of the distribution has only a nominal impact on the derivation of Equation (28), the associated entropy can be calculated accordingly. This is not as outlandish of a suggestion as might appear at first glance: the Booij entropy expression in terms of second moments of distribution functions, Equation (28), has been shown to be consistent with many polymer rheological models derived and employed during the past 50 years [8,23,24]. The accuracy of this approximation, assuming it actually is one, can be examined via NEMD simulation; the results of such an examination were recently reported [25] and are extended and elaborated upon below.

Once the correct entropy at the mesoscopic level has been ascertained, the task turns to calculation of the corresponding Helmholtz free energy (or Gibbs free energy if working in the NpT ensemble). Typically, the change in free energy is expressed through the Legendre transformation (at constant temperature) ΔA=ΔU−TΔS. In the Rouse Model, it is assumed that ΔU=0, since the internal energy of all available configurations is taken to be the same [17,18]. For the longer modes, this assumption is reasonable, since the interatomic energetic potentials act within a localized area surrounding individual atoms on the order of a Kuhn segment, something like 25 Å. Hence the long modes, corresponding to length scales spanning many Kuhn segments, retain statistically driven configurational states wherein any applicable energetic interactions are effectively relegated to the local neighborhoods of individual Kuhn segments that make up the global molecular segments represented by the springs of the Rouse Model. Consequently, extension of the springs does not necessarily imply a change in the energetics of the Kuhn segments that constitute them. However, for the shorter time-scale modes, which effectively describe smaller segments of the chain, energetic effects can become significant as the length scale of the effective submolecular segment approaches that of several Kuhn steps. (Recall that the Rouse Model quantifies polymer chains in solution at infinite dilution. It is known that in dense melts favorable energetic interactions arise for highly stretched and oriented molecules as they align with each other side-to-side, thereby reducing the intermolecular energy [14,26,27,28].) A sensible question to ask is, at what level of chain discretization do the energetic effects become important in determining the free energy of the corresponding molecular segment? A possibly simplistic answer to this question is: at the level where the distribution of the segment’s spanning vector becomes non-Gaussian under equilibrium conditions; i.e., when a purely random statistical response is not adequate for quantifying the spanning vector distribution, energetic effects are likely to be distorting it. (This will be illustrated below in Section 4.2.) Note that this condition that all submolecular chain segments obey Gaussian statistics is exactly that which was employed by Rouse in his original derivation [17].

#### Maxwell Relations at the Mesoscale

Before moving on to a discussion of entropy at the atomic-length scale, it is interesting to examine the Maxwell relationships corresponding to the Rouse Model. In the NVT ensemble, the free energy of the liquid can be expressed as function $A(T,V,C_{k})$, with $C_{k}$ denoting the second moments of the individual Rouse modes. As discussed above, Maxwell relations can be derived in terms of the components of the second moments and their corresponding conjugate variables (i.e., deformation potentials); however, the individual modes are independent of each other and relationships involving mixed partial derivatives of pairs of differing mode components identically vanish. Defining the deformation potential of mode *k* as $Z_{\alpha \beta }\equiv \partial A/\partial C_{\alpha \beta }$, the Maxwell relationships for the Rouse Model are equivalent to Equation (10):

(29) $$\frac{\partial Z_{\alpha \beta }}{\partial C_{\gamma \epsilon }}=\frac{\partial Z_{\gamma \epsilon }}{\partial C_{\alpha \beta }}\;\;.$$

(Note that we have dropped the mode number indicator *k* for notational convenience since the following expressions apply to each individual mode.) The contribution to the free energy of the Rouse Model of the second moments of the individual mode distributions was derived by Booij [18] according to Equation (28) (note that the Rouse Model assumes that internal energy is not a function of the fluid’s internal microstructure):

(30) $$A=\frac{Nk_{B}T}{2}\left(\sum_{k}\mathrm{tr}\;C_{k}-\sum_{k}\ln|\det\;C_{k}|\right)\;\;.$$

For each mode,

(31) $$\frac{\partial A}{\partial C_{\alpha \beta }}=\frac{Nk_{B}T}{2}\left(\delta_{\alpha \beta }-C_{\beta \alpha }^{-1}\right)=Z_{\alpha \beta }\;\;,$$

but it should be noted that both C and $C^{-1}$ are symmetric, the former by definition (see e.g., Equation (6)) and the latter as an application of linear algebra. The Maxwell relations of Equation (29) then become

(32) $$\frac{\partial C_{\alpha \beta }^{-1}}{\partial C_{\gamma \epsilon }}=\frac{\partial C_{\gamma \epsilon }^{-1}}{\partial C_{\alpha \beta }}\;\;,$$

however,

(33) $$\frac{\partial C_{\alpha \beta }^{-1}}{\partial C_{\gamma \epsilon }}=-C_{\alpha \eta }^{-1}\;\frac{\partial C_{\eta \zeta }}{\partial C_{\gamma \epsilon }}\;C_{\zeta \beta }^{-1}=-C_{\alpha \gamma }^{-1}C_{\epsilon \beta }^{-1}\;\;,$$

such that the Maxwell relation of Equation (29) becomes

(34) $$C_{\alpha \gamma }^{-1}C_{\epsilon \beta }^{-1}=C_{\gamma \alpha }^{-1}C_{\beta \epsilon }^{-1}\;\;,$$

which is identically satisfied since $C^{-1}$ is symmetric. Hence, the free energy of the Rouse Model, as derived in terms of the second moments of the distributions of the individual relaxation modes [18], duly satisfies Maxwell relations of the form of Equation (29).

Expressing the overall free energy of the Rouse liquid according to Equation (30) as

(35) $$A\left(T,V,C_{k}\right)=A_{0}\left(T,V\right)+\frac{Nk_{B}T}{2}\left(\sum_{k}\mathrm{tr}\;C_{k}-\sum_{k}\ln|\det\;C_{k}|\right)\;\;,$$

the standard thermodynamic relation [8] S=−∂A/∂T yields

(36) $$S\left(T,V,C_{k}\right)=S_{0}\left(T,V\right)-\frac{Nk_{B}}{2}\left(\sum_{k}\mathrm{tr}\;C_{k}-\sum_{k}\ln|\det\;C_{k}|\right)\;\;.$$

This expression is consistent with the entropy expression of Booij for the internal mode contributions to the liquid entropy, Equation (28). (Note that $A_{0}$ and $S_{0}$ can be viewed as the solvent free energy and entropy, respectively). Recognizing the thermodynamic pressure as [8]$p=-\partial A/\partial V=-\partial A_{0}/\partial V$, Equations (31), (35) and (36) admit proof of the remaining Maxwell relationships,

(37) $$-\frac{\partial S}{\partial C_{\gamma \epsilon }}=\frac{\partial Z_{\gamma \epsilon }}{\partial T}\;\;,\;\;\;\frac{\partial S}{\partial V}=\frac{\partial p}{\partial T}\;\;,\;\;\;\frac{\partial p}{\partial C_{\gamma \epsilon }}=\frac{\partial Z_{\gamma \epsilon }}{\partial V}\;\;.$$

Hence, the Rouse Model satisfies all thermodynamic requirements consistent with the extended Gibbs relationship of Equation (7).

Thermodynamic quantities such as the heat capacity at constant volume can also be obtained using the internal energy function expressed in terms of temperature and volume. The symbol $U^{\prime }\left(T,V\right)$ is used here for internal energy rather than U(S,V) to emphasize that the internal energy function is expressed in terms of temperature instead of entropy.) $U^{\prime }\left(T,V\right)=A\left(T,V,C_{k}\right)+T\;S\left(T,V,C_{k}\right)$, such that

(38) $$\frac{\partial U^{\prime }}{\partial T}=C_{v}=-S+S+T\;\frac{\partial S}{\partial T}\;\;;$$

however, with *S* given by Equation (36),

(39) $$\frac{\partial S}{\partial T}=\frac{\partial S_{0}}{\partial T}=\frac{C_{v}}{T}\;\;.$$

Hence, the heat capacity at constant volume does not depend on the internal relaxation modes of the Rouse Model. This is intuitively expected since $U^{\prime }$ is not a function of $C_{k}$, which is a result of the assumption of Gaussian chain statistics; i.e., the thermodynamic system is governed solely by random configurational probabilities, which implicitly deny the existence of energetic interactions between the submolecular segments.

### 3.2. Calculation of Entropy at the Atomic Scale

Some issues with calculating entropy at the mesoscale were discussed above at length. Entropy can be calculated at various levels of coarse-graining via counting microstates of the full molecule or statistically relevant but arbitrarily smaller subunits of the molecular chain. How consistent these various entropy calculations are with each other remains to be seen. Part of the problem with calculating the entropy is the rather large size of the constituent macromolecules relative to their internal atomic architectural units. In principle, however, one might expect that entropy could be calculated at an atomic level by summing up and averaging over the entropy calculated in local environments within the bulk fluid, in essentially the same manner as the stress is calculated locally. In most NEMD simulations, long-range corrections to the Lennard–Jones interaction potential are neglected such that stress is effectively calculated within regions of radius less than about 25 Å and then summed over the entire simulation cell and averaged over time. The entropy should also be calculable in the same fashion, although the computations are unmanageable unless (possibly severe) approximations are assumed. Once one accepts this proposition, entropy can be calculated at the local atomic scale, generally completely within one or two Kuhn steps of the overall polymer chain. Thus, in some sense, the two perspectives are rather contradictory: at the mesoscale, what goes on within the Kuhn segments is irrelevant to the entropy calculation, whereas at the atomic scale, that is all there is.

In principle, entropy can be calculated directly from the phase space variables of the material, $\Gamma \left(x^{N},\;p^{N}\right)$, where $x^{i}$ is the position vector of particle *i*; $p^{i}$ is its momentum vector; and *N* is the number of atoms or molecules. The probability that the *N* molecules occupies a given configurational state of phase space can then be expressed by

(40) $$f_{N}\;\prod_{i=1}^{N}d\Gamma^{i}\;\;,$$

where $f_{N}\left(x^{N},\;p^{N}\right)$ is the probability distribution function in 6N-dimensional phase space, and $d\Gamma^{i}$ is the volume element of phase space associated with particle *i*. An expression for the entropy based on phase space was developed by Green [29] (Herbert Sydney, known today for the BBGKY hierarchy and Born–Green reciprocity) as (phase-space volume here is expressed in terms of the coordinate volume element of particle 1 and the phase-space volume of the other particles, plus the momentum space of the first particle, because $f_{N}$ depends only on the relative positions of the particles)

(41) $$S=-\frac{k_{B}}{N!}\;\int \int_{V}f_{N}\;\ln\;f_{N}\;dx^{1}d\Gamma^{N-1}\;\;.$$

Shortly thereafter, Stratonovich [30] developed an expression for entropy based only on the configurational elements of phase space (note that the sum in this expression is the limit of a finite sum for a system of *N* particles),

(42) $$S=-k_{B}\;\sum_{i=0}^{\infty }\frac{1}{i!}\;\int_{V}e_{i}\left(\left[i\right]\right)\;\ln\;e_{i}\left(\left[i\right]\right)\;dx^{i}\;\;,$$

where $e_{i}\left(\left[i\right]\right)\;dx^{i}$ is the probability of simultaneous occurrence that there are exactly *i* particles in *V* and that they occupy the configuration $\left[i\right]=\left(x^{1},p^{1},\ldots ,x^{i},p^{i}\right)$. Nettleton and Green [31] (a different Green, Melville Saul, known principally for the Green–Kubo relations) applied several approximations to this equation; in particular, they expressed it in terms of the radial distribution function, g(r), and considered only two-body correlations, ultimately deriving an approximate expression for the excess (relative to an ideal gas) configurational entropy per particle (note that by the nature of the applied approximations, this expression is generally valid only for a rarified gas),

(43) $$S_{i}=-2\pi \rho_{N}k_{B}\int_{0}^{\infty }\left[g^{i}\left(r\right)\ln g^{i}\left(r\right)-g^{i}\left(r\right)+1\right]r^{2}\;dr+\ldots \;\;,$$

where $g^{i}\left(r\right)$ is the radial distribution function (RDF) centered at the *i*-th particle; $\rho_{N}$ is the overall particle number density; and *r* is the spatial distance coordinate emanating from particle *i*.

Recognizing the approximate nature of this expression for dense liquids, but intrigued by the possibility of calculating the entropy associated with crystallization via molecular simulation using RDFs, Piaggi et al. [32,33] recently presented a method by which Equation (43) could be applied to study the crystallization of sodium and aluminum from the liquid phase into body-centered cubic and face-centered cubic lattices, respectively. Although not exact for these systems, the authors argued that roughly 90% of the configurational entropy of these metal elements was captured adequately enough by Equation (43) to accelerate the crystallization by enhanced entropy sampling, and thereby the rate of nucleation events without biasing the outcome in any particular direction. Nafar Sefiddashti et al. [34] applied this method to the study of flow-enhanced nucleation and crystallization of an entangled linear polyethylene melt undergoing elongational flow, demonstrating that the particle entropy expression of Equation (43) (coupled with an analogous expression for enthalpy) could provide a robust, thermodynamic-like methodology for monitoring nucleation events and crystallization during molecular simulations of polymer melts and solutions. The premise of this research, however, was that nucleation and crystallization would manifest as a large change in entropy regardless of its exact value, such that even an approximate expression would provide adequate indication of an incipient phase change. Such a premise appeared to be validated, but possibly any approximation errors were of sufficiently small magnitude not to affect the rather large changes in entropy induced by the phase change. The question remains, however, whether such reasoning can equally be extended to polymeric melts and solutions undergoing flow; i.e., can Equation (43) be used to calculate a thermodynamically relevant nonequilibrium configurational entropy change associated with the application of flow relative to the quiescent liquid state (even though the entropy of the quiescent state is largely unknown and the part that is known is only approximate)? This question was not investigated in the prior work [25].

## 4. Methodology

### 4.1. Simulation Methods and Systems

The simulated system is a monodisperse, linear, entangled C_1000_H_2002_ PE melt undergoing steady-state planar Couette flow (PCF) and planar extensional flow (PEF) over a wide range of deformation rates. The nonequilibrium molecular dynamics (NEMD) PCF simulations were performed over the range 0<Wi≤1170, where the Weissenberg number, Wi, is defined as $Wi\equiv \dot{\gamma }\tau_{d}$ with $\dot{\gamma }$ being the shear rate and $\tau_{d}=5270$ ns being the disengagement time of the polymer under quiescent conditions [13]. The PEF simulations were performed over the range 0<De≤15, where $De\equiv \dot{\varepsilon }\tau_{R}$ is the Deborah number; $\dot{\varepsilon }$ is the extension rate; and $\tau_{R}=137$ ns is the Rouse relaxation time of the polymer at equilibrium [13]. Nonequilibrium molecular dynamics (NEMD) simulations were performed in the *NVT* ensemble at 450 K and the density 0.766 g/cm^3^. The modified Siepmann–Karaborni–Smit (SKS) potential model [35,36] was used to determine the energetic interactions between the atomic units of the PE molecules, which were viewed as united atoms composed of a carbon atom and its bound hydrogen atoms [35]. Information about the simulation cell dimensions, flow field parameters, and the total number of atoms are provided in Table 1.

The SKS potential model [35,36] consists of a harmonic bond-stretching potential acting between adjacent united atoms [36], a bond-bending potential maintaining the angle between successive bonds, a torsional energy quantifying the rotation of the bonds around individual atomic units, and intramolecular and intermolecular Lennard–Jones (LJ) potentials acting, respectively, between atoms on the same PE molecule separated by more than three bonds and atoms on separate molecules [35]. The intermolecular and intramolecular nonbonded energetic interactions in this model are given by a 12–6 LJ potential,

(44) $$U_{LJ}\left(r_{ij}\right)=4\epsilon_{ij}\left[{\left(\frac{\sigma_{ij}}{r_{ij}}\right)}^{12}-{\left(\frac{\sigma_{ij}}{r_{ij}}\right)}^{6}\right]\;\;,$$

where $r_{ij}$ is the distance between atoms *i* and *j*. Note that the intramolecular energy is only applied to atoms separated by more than three bonds. The energetic parameters ($\epsilon_{i}/k_{B}$) were 47 K for CH_2_ units and 114 K for CH_3_ units [35]. The distance parameters were $\sigma_{CH_{2}}=\sigma_{CH_{3}}=3.93$ Å for both CH_2_ and CH_3_ units. Lorentz–Berthelot mixing rules were used to calculate the interaction parameters between atomic units *i* and *j*, such that

(45) $$\begin{array}{cc} \epsilon_{ij} & ={\left(\epsilon_{i}\epsilon_{j}\right)}^{1/2}\;\;,\\ \sigma_{ij} & =\frac{\sigma_{i}+\sigma_{j}}{2}\;\;. \end{array}$$

A cut-off distance $r_{c}=2.5\sigma$_CH_2__ was employed for all LJ potentials.

The bond-stretching interaction energy is described by a harmonic potential function,

(46) $$U_{str}\left(l\right)=\frac{1}{2}k_{l}{\left(l-l_{eq}\right)}^{2}\;\;,$$

where *l* is the distance between adjacent atoms of the same molecule. The bond-stretching constant was $k_{l}/k_{B}$ = 452,900 K/Å^2^, and the equilibrium bond length was $l_{eq}=1.54$ Å [36]. The bond-bending potential energy also was governed by a harmonic potential function of the form

(47) $$U_{ben}\left(\theta \right)=\frac{1}{2}k_{\theta }{\left(\theta -\theta_{eq}\right)}^{2}\;\;,$$

where θ is the angle formed between three successive atoms. The bond-bending constant in this equation was $k_{\theta }/k_{B}$ = 62,500 K/rad^2^, and the equilibrium angle was $\theta_{eq}=114^{\circ }$.

The bond-torsional energy was expressed as

(48) $$U_{tor}\left(\phi \right)=\sum_{m=0}^{3}a_{m}{\left(\cos\phi \right)}^{m}\;\;,$$

where the bond-torsional constants were $a_{0}/k_{B}=1010$ K, $a_{1}/k_{B}=-2019$ K, $a_{2}/k_{B}=136.4$ K, and $a_{3}/k_{B}=3165$ K. This potential was used on atoms of the same chain that were separated by three bonds. Using the physical parameters described above, the maximum possible extension of the C_1000_H_2002_ chains is approximately 1290 Å when all bond dihedral angles are in the *trans* configuration and all bonds and angles are at their equilibrium positions.

Nonequilibrium molecular dynamics simulations were performed using the p-SLLOD equations of motion with a Nosé-Hoover thermostat [37,38,39,40,41] implemented within the Large-scale Atomic/Molecular Massively Parallel Simulator (LAMMPS) [42] computing environment. The full set of evolution equations for the system are

(49) $$\begin{array}{cc} \; & {\dot{q}}_{ia}=\frac{p_{ia}}{m_{ia}}+q_{ia}\cdot \nabla u\;\;,\\ \; & {\dot{p}}_{ia}=F_{ia}-p_{ia}\cdot \nabla u-m_{i}q_{ia}\cdot \nabla u\cdot \nabla u-\frac{p_{\xi }}{Q}p_{ia}\;\;,\\ \; & \dot{\xi }=\frac{p_{\xi }}{Q}\;\;,\\ \; & \dot{p_{\xi }}=\sum_{i}\sum_{a}\frac{p_{ia}^{2}}{m_{ia}}-dN_{a}k_{B}T\;\;,\\ \; & Q=dN_{a}k_{B}T\tau_{t}^{2}\;\;. \end{array}$$

In this set of equations, $m_{ia}$, $q_{ia}$, $p_{ia}$, and $F_{ia}$ are the mass, position, momentum, and force vectors of atom *a* in molecule *i*, respectively. ξ and $p_{\xi }$ represent the coordinate-like and momentum-like variables of the thermostat, respectively. The symbol *d* represents the spatial dimensionality (3), *Q* is the thermostat mass parameter, and $\tau_{t}$ is the relaxation time of the thermostat.

The local streaming velocity profile imposed on the system is quantified by the velocity gradient tensor. In PCF, flow is applied in the *x* direction with the gradient in the *y* direction. In PEF, elongation occurs in the *x* direction with compression in the *y* direction. ∇u is the velocity gradient tensor, which for PCF takes the form

(50) $$\nabla u=\left(\begin{array}{ccc} 0 & 0 & 0\\ \dot{\gamma } & 0 & 0\\ 0 & 0 & 0 \end{array}\right),$$

and for PEF,

(51) $$\nabla u=\left(\begin{array}{ccc} \dot{\epsilon } & 0 & 0\\ 0 & -\dot{\epsilon } & 0\\ 0 & 0 & 0 \end{array}\right).$$

Boundary conditions were periodic at all box surfaces with a simulation box deforming in the *x* direction for PCF, and a deforming box, elongating in the *x* direction and compressing in the *y* direction, for PEF. The NEMD equations were integrated using the reversible-Reference System Propagator Algorithm, r-RESPA, with two different time steps: for all PCF simulations and PEF simulations where De<0.5, the long time step was 4.70 fs, which was used for the slowly varying nonbonded LJ interactions, and the short time step was 1.175 fs (one-fourth of the long time step) for the rapidly varying forces including bond-bending, bond-stretching, and bond-torsional interactions. For PEF simulations where De>0.5, the long time step was 2.35 fs, and the small time step was 0.47 fs. The relaxation time of the thermostat was set equal to 100 times the long time step for all simulations. NVT simulations were initiated from an equilibrated configuration under quiescent conditions at a specific value of Wi or De (≠0) and run until all statistical indicators revealed that a steady-state condition had been achieved. For the PEF simulations, the Kraynik–Reinelt periodic boundary conditions were employed to allow for unrestricted simulation time of the deforming simulation box undergoing PEF over the full range of imposed strain rates [43]. Accordingly, the simulation cell was deformed by elongating in the flow direction (*x*) and shrinking in the compression direction (*y*) while rotating to maintain the associated periodicity of the boundaries. After each 0.9624 Hencky strain units, the cell was transformed back to its original dimensions and orientation.

### 4.2. Calculation of Entropy at the Mesoscale

To calculate entropy at the mesoscale, one must first select a (or more than one) coarse-grained variable to represent the configurational microstate of the molecules within the liquid. The larger the chosen variable, the more degenerate its distinct microstates will be, and the longer the length and time scales it will possess. Several levels of coarse-graining will be examined below, but the methodology is presented in terms of the end-to-end vector, R, such that the entropy change relative to the quiescent liquid is given by Wall’s Equation (25). Computation of the entropy change from NEMD simulation via Equation (25) was performed using a recently developed method by Edwards et al. [25]. The entropy change was computed by evaluating the integrand, $I=\psi \;\ln\left(\psi_{eq}/\psi \right)$, which required the calculation of the 3-d equilibrium, $\psi_{eq}$, and nonequilibrium, ψ, probability distribution functions (PDFs) of configurational microstates based on the individual molecule R. Density functions were calculated by binning the ensemble of end-to-end vectors consisting of all chains at steady-state and then normalizing the resultant histograms—see below for details regarding the choice of a suitable number of bins. Note that the integration interval is theoretically (−∞,+∞) for all three components of R; however, the integration is more practically performed within the range $\left[-R_{\max},R_{\max}\right]$, where $R_{\max}\equiv {∥R∥}_{\max}$ is the theoretical maximum chain end-to-end distance; i.e., a chain with all dihedral angles occupying *trans* conformations. Even within this narrow range, the probability densities for many values of R are negligibly small. Especially, $\psi_{\mathrm{eq}}$ and ψ at low and intermediate deformation rates effectively go to zero as the components of R approach $R_{\max}$. This situation is aggravated further when the PDFs are calculated from molecular dynamics simulations because of the limited accuracy due to the restricted ensemble available. Such zero probabilities in certain bins are problematic when evaluating the integrand of Equation (25), as they appear as arguments of a logarithm function.

These numerical problems can be partially avoided by algebraic manipulations of the integrand *I*:

(52) $$\begin{array}{c} I=\psi \;\ln\left(\frac{\psi_{\mathrm{eq}}}{\psi }\right)=\psi \ln\left(\psi_{\mathrm{eq}}\right)-\psi \ln\left(\psi \right). \end{array}$$

The problem of ${\ln\left(\psi \right)|}_{\psi =0}$ could be handled by using L’Hôpital’s rule to show that $\lim_{\psi \to 0}\psi \ln\left(\psi \right)=0$. Hence, the second term of Equation (52) vanishes for ψ→0; however, the same does not apply to the first term. Specifically, $\lim_{\psi_{\mathrm{eq}}\to 0}\psi \ln\left(\psi_{\mathrm{eq}}\right)$ is undefined. Therefore, zero values of the equilibrium PDF, $\psi_{\mathrm{eq}}$, should be avoided altogether.

**Figure 2:**
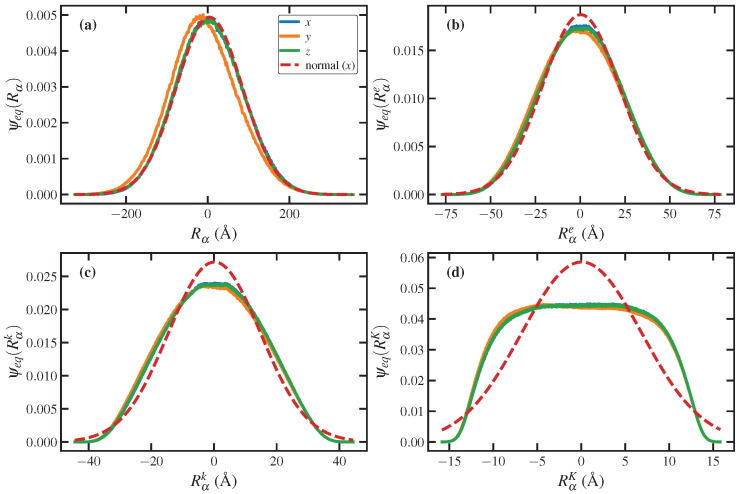
Probability distributions of the components of segmental end-to-end vectors for the C_1000_H_2002_ liquid at equilibrium: (**a**) $R_{\alpha }$ represents the α component of the overall molecule end-to-end vector component, (**b**) $R_{\alpha }^{e}$ is the entanglement strand vector component α, (**c**) the kink strand spanning vector component is $R_{\alpha }^{k}$, and (**d**) the Kuhn segment vector component is $R_{\alpha }^{K}$. Note that the red dashed line represents a corresponding normal (Gaussian) distribution for comparison.

As discussed above, zero values of $\psi_{\mathrm{eq}}$ in bins at the extreme ends of the R range are inevitable if one relies only on values obtained from simulation. However, the equilibrium distribution configuration of flexible polymeric liquids can be reliably assumed to be Gaussian. Indeed, the C_1000_H_2002_ liquid simulated in the present study was shown to exhibit Gaussian PDFs under quiescent and weak flow conditions [13,44]. In Equation (52), $\psi_{\mathrm{eq}}$ is the PDF for the chain end-to-end vector (not its magnitude), and hence it is a multivariate distribution, with *x*, *y*, and *z* components of the end-to-end vector as independent variables. $\psi_{\mathrm{eq}}$ can be estimated very well with a Gaussian distribution function (see panel (a) of Figure 2) expressed as

(53) $$\psi_{\mathrm{eq}}=\frac{1}{\sqrt{{\left(2\pi \right)}^{3}\det\Sigma }}\exp\left(-\frac{1}{2}{\left(R-\mu \right)}^{T}\cdot \Sigma^{-1}\cdot \left(R-\mu \right)\right),$$

where $\mu^{T}=(\langle R_{x}\rangle ,\langle R_{y}\rangle ,\langle R_{z}\rangle$) is the mean vector composed of ensemble averages of components of the end-to-end vector, $R_{\alpha }$, and $\Sigma_{\alpha \beta }=\mathrm{Cov}\left(R_{\alpha },R_{\beta }\right)$ is the covariance matrix. Note that this estimate is only applied when $\psi_{\mathrm{eq}}$ happens to be zero in particular bins on the extreme edges of the variable range, as discussed above. $\psi_{\mathrm{eq}}$ obtained from Equation (53) can become indefinitely close to zero but never exactly zero; therefore, this equation mitigates the numerical error of $\psi \ln(\psi_{\mathrm{eq}})$ when $\psi_{\mathrm{eq}}=0$ within certain bins of the simulation. After evaluation of *I*, the triple integral of Equation (25) can be computed via numerical integration. The trapezoidal rule was employed using the same number of sample points as the number of bins of the PDFs used in the calculation of *I* in each direction.

In the preceding discussion, the individual chain configurations and the associated nonequilibrium entropy were quantified using the end-to-end vector, R, and its 3-d probability distribution functions (PDFs). As such, much of the atomistic detail of the chain was neglected from the overall description. Where and when such action is warranted and allowable is irrelevant at present. The necessary detail to be included in the formulation depends on the objectives of the problem and the relevant time and length scales of the observer, as discussed above. Other variables (discretizations) of the individual molecules can also be considered where each chain is partitioned into a number of segments of various length, representing different levels of coarse-graining.

Three additional chain variables are defined at various levels of coarse-graining in addition to that of the end-to-end vector discussed above, as follows. The first was based on the entanglement length of a typical linear PE; i.e., the average length of a polymer segment between entanglement junctions at equilibrium. Experimentally, the entanglement molecular weight is known to be 1150 g/mol [45], which corresponds to an average number of entanglements per chain for the C_1000_H_2002_ liquid simulated herein of approximately 12.2. Furthermore, using the Z1 code [46,47], the average number of entanglements per chain was similarly calculated as 12.9 directly from equilibrium simulation. This results in a chain composed of 14 segments (or entanglement strands), each representing the average distance between entanglement points at equilibrium, 37.3 Å. As such, $R^{e}$ was defined as the vector spanning a segment from one end to the other, and then 3-d PDFs, $\psi (R_{\alpha }^{e})$, were computed in terms of this vector directly from the NEMD simulations. Furthermore, second moments of the PDFs were also calculated using a definition analogous to that of the main text, $C^{e}=\langle 3\;R^{e}R^{e}\rangle /{\left(R_{eq}^{e}\right)}^{2}$. The next discretization variable was based on the number of chain kinks (26) as computed by the Z1 code, resulting in 27 chain segments of average length at equilibrium of 25.6 Å. PDFs $\left(\psi (R_{\alpha }^{k})\right)$ based on this discretization were also calculated from the simulations, as well as their second moments $\left(C^{k}=\langle 3\;R^{k}R^{k}\rangle /{\left(R_{eq}^{k}\right)}^{2}\right)$, using the segment spanning vector $R^{k}$. Lastly, a discretization variable was defined based on the Kuhn segment of the PE melt as composed of a string of 13 adjoined carbon atoms of contour length 15.6 Å (as calculated from the equilibrium simulation). Here, the vector $R^{K}$ spanned the distance between the ends of each Kuhn segment. This yielded a chain of 83 segments, each of which can only be activated under strong flows and which incorporates dynamics occurring within the first several nearest neighbor shells surrounding individual atoms. PDFs $\left(\psi (R_{\alpha }^{K})\right)$ and second moments $\left(C^{K}=\langle 3\;R^{K}R^{K}\rangle /{\left(R_{eq}^{K}\right)}^{2}\right)$ based on the Kuhn segments were defined and calculated as for the other discretizations, as discussed above.

Under equilibrium conditions, the overall chain end-to-end vector exhibits a fully 3-d probability distribution that is essentially Gaussian, as illustrated in panel (a) of Figure 2. The PDF based on the vector spanning the entanglement strands also remains approximately Gaussian—see panel (b). However, the PDF based on the number of kinks (panel c) and Kuhn length (panel d) are decidedly non-Gaussian even under equilibrium conditions. Note that the bottom panel indicates that the lengths of the individual Kuhn segments are all fairly consistent (but with random orientations). Also note that the flat distribution begins to fall off at about $|R_{\alpha }^{K}|>10$ Å and asymptotes to zero for $|R_{\alpha }^{K}|>15$ Å, the latter value being close to the computed Kuhn length. These non-Gaussian PDFs require additional care when computing the entropy according to Wall’s 3-d entropy equation.

A Gaussian approximation for $\psi_{\mathrm{eq}}$ begins to break down for segments as long as kink strands are shorter—see Figure 2c. Therefore, using Equation (53) might seem irrelevant in such cases; however, this distribution is only applied to unoccupied bins at the extreme edges of the variable range, where it remains approximately valid for $P\left(R_{\alpha }^{k}\right)$, but it is a rather poor approximation at the edges for $P\left(R_{\alpha }^{K}\right)$ (see panel (d) of Figure 2). It should be emphasized again that a Gaussian approximation for $\psi_{\mathrm{eq}}$ is only necessary when the occupancy of particular bins happens to be zero on the extreme edges of the variable range. It is evident from Figure 2 that for these extreme edges, most of the PDFs (except for those associated with the Kuhn step) obtained from the MD results approach a Gaussian distribution again and asymptotically go to zero. Therefore, using Equation (57), as discussed below, would be reasonably accurate for most levels of chain discretization but even so should only be used when absolutely necessary. This issue with unoccupied bins at the edges of the variable range is the major source of error in computing entropy using Wall’s equation.

**Figure 3:**
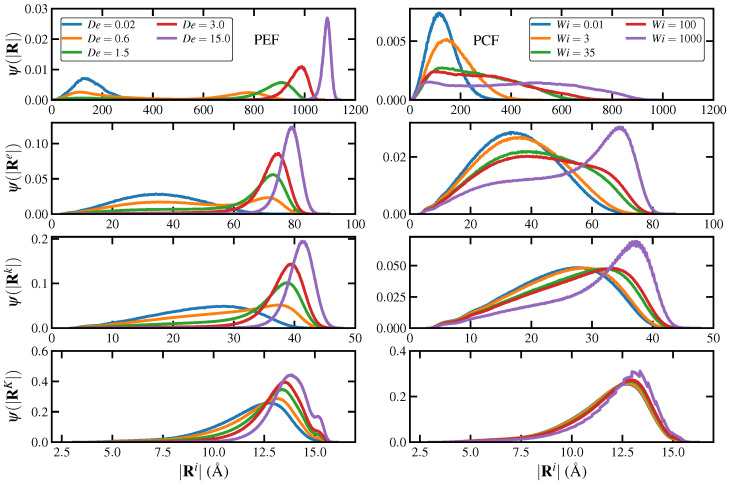
Probability distributions of the magnitude of the segmental spanning vector of the C_1000_H_2002_ liquid in PEF (column 1) and PCF (column 2) at various values of dimensionless strain rate. From top to bottom: overall molecule end-to-end vector, the entanglement strand vector, the kink strand vector, and the Kuhn segment vector. The $R^{i}$ on the abscissas represents any of the corresponding four relevant variables as depicted on the ordinates.

Note that in previous simulation studies, the Kuhn length of linear monodisperse polyethylenes was reported as approximately 16 Å. Indeed, for the C_1000_H_2002_ liquid simulations reported herein, the Kuhn length calculated at equilibrium was $\ell_{K}=\langle R^{2}\rangle /R_{\max}$ = 15.6 Å [13,44], where R in this expression is the overall chain end-to-end vector, and $R_{\max}$ is the contour length (maximum chain extension). Based on the statistics of a random-walk polymer chain, $R_{\max}=N_{k}\ell_{K}$=1290 Å, where $N_{K}$ is the number of Kuhn segments; hence, $N_{K}$ works out to be about 83 in the present simulations. The discretization at the Kuhn level was thus based on subdividing the overall macromolecule into 83 segments, each composed of 13 adjoined carbon atoms with a contour length of 15.6 Å. However, as measured directly from the equilibrium simulations, because the individual Kuhn segments are not fully stretched but are rather bent, the vector spanning the ends of the segment has a mean magnitude of approximately 11.8 Å, as evident in panel (d) of Figure 2 and the bottom panels of Figure 3 at the lowest De (0.02) and Wi (0.01) values. This discrepancy is due to the fact that the molecules in the simulation are energetically forbidden from occupying the same space by the hard repulsive 12-6 LJ potential. Therefore, instead of a random-walk model, a self-avoiding-walk model is more appropriate. In 3-d space, it is known that [48,49]${\langle R^{2}\rangle }^{1/2}=\ell_{K}N_{K}^{0.586}$. Since ${\langle R^{2}\rangle }^{1/2}=142$ Å, for 83 segments the segment spanning vector should have a mean magnitude of 10.7 Å. This value is reasonably consistent with the value of 11.8 reported above. This is the effective persistence length of the PE macromolecules simulated herein.

The outcome of the numerical integration of Wall’s equation can be influenced by the number of bins employed in computing the PDFs ψ and $\psi_{\mathrm{eq}}$. On the one hand, a very small number of bins could lead to losing important features of the distributions and hence large errors in the integrands and corresponding integrals that are calculated based on them. On the other hand, an excessively large number of bins can lead to noisy PDFs (due to the limited number of data points available from NEMD), which again introduces significant errors when the integrals are calculated using numerical methods. Furthermore, such large bin numbers require tremendous amounts of memory, especially when evaluating segmental PDFs, which can easily exceed contemporary computational resources. Statisticians commonly employ various rules of thumb to determine an appropriate number of bins (or bin width) based on data size and the shape of the distribution. In this work, Doane’s formula was used to obtain an initial estimate for a suitable number of bins. Doane’s formula for the number of bins, $n^{D}$, is expressed as [50]

(54) $$\begin{array}{ccc} & & n^{D}=1+\log_{2}\left(n_{d}\right)+\log_{2}\left(1+\frac{\left|g_{1}\right|}{\sigma_{g_{1}}}\right)\;, \end{array}$$

(55) $$\begin{array}{ccc} & & g_{1}=\mathrm{mean}\left[{\left(\frac{x-\mu }{\sigma }\right)}^{3}\right]\;, \end{array}$$

(56) $$\begin{array}{ccc} & & \sigma_{g_{1}}=\sqrt{\frac{6(n_{d}-2)}{\left(n_{d}+1\right)\left(n_{d}+3\right)}}\;\;, \end{array}$$

where $n_{d}$ is the number of data points in the sample, μ is the mean value, and σ is the standard deviation. $g_{1}$ is the third standardized moment representing the skewness of the data. This formula was used for various components of the overall chain or chain segment-spanning vectors to obtain an approximate number of bins, $n_{\alpha }^{D}$, for each component α. The exact number of bins in each direction was chosen as $n_{\alpha }=pn_{\alpha }^{D}$, where *p* was an arbitrary coefficient of order unity. Note that p>1 provides a more conservative estimate of the number of bins than that of Doane’s expression. In this work, p=1 was used for all cases, except for the PDFs of the end-to-end vector for the PEF simulations at De≥3 where p=3 was used. Overall, for the simulations of the present study, p=1–3 resulted in roughly 30 to 100 bins, which led to consistent values of the entropy.

As discussed above, the Gaussian PDF, Equation (53), was used under equilibrium conditions in situations where $\psi_{\mathrm{eq}}$ turned out to be zero in certain bins from the NEMD data. Note that at equilibrium conditions, various components of the overall chain end-to-end vector or chain segment-spanning vector (at least down to the level of Kuhn segments) are practically independent and uncorrelated. This leads to some simplifications in Equation (53); namely, off-diagonal components of the covariance matrix, Σ, vanish because of this lack of correlation. Hence, Σ is practically a diagonal matrix. Calculation of the inverse and determinant of a diagonal matrix is significantly computationally cheaper and more straightforward. Note that the diagonal elements are the variances in the *x*, *y*, and *z* components of the relevant spanning vectors. Furthermore, in the case of using Equation (53) as $\psi_{\mathrm{eq}}$, it was more convenient and efficient to insert directly $\ln(\psi_{\mathrm{eq}})$ in Equation (52); i.e.,

(57) $$\ln\left(\psi_{\mathrm{eq}}\right)=\ln\left(\frac{1}{\sqrt{{\left(2\pi \right)}^{3}\det\Sigma }}\right)-\frac{1}{2}{\left(R-\mu \right)}^{T}\cdot \Sigma^{-1}\cdot \left(R-\mu \right)\;\;,$$

when expressed in terms of the end-to-end vector. A similar expression can be written for any of the chain discretizations discussed above. This simplification was useful in avoiding large numbers in the evaluation of the exponential term of Equation (53).

Booij [18] derived an expression for the entropy change, (28), of the Rouse Model of a dilute polymer solution based on the assumption that the $\psi_{k}$ and $\psi_{k,eq}$ were Gaussian distributions. It is therefore not to be expected that such an expression would play any role in evaluation of the entropy of entangled PE melts such as C_1000_H_2002_. Nevertheless, it was demonstrated recently that the general form of this expression turns out to be very useful if the second moments of the Gaussian distributions assumed by Booij are replaced with the second moments of the C_1000_H_2002_ liquid taken directly from the NEMD simulations [25]. The entropy changes according to this method, $\Delta S_{sim}^{i}$, are calculated using an expression of similar form to Booij’s entropy of Equation (28),

(58) $$\Delta S_{sim}^{i}=-\frac{N_{i}k_{B}}{2}\left[\mathrm{tr}\left(C_{sim}^{i}-\delta \right)-\ln|\det\;C_{sim}^{i}|\right]\;\;,$$

where $N_{i}$ is the total number of chain segments for discretization *i*, and $C_{sim}^{i}$ is the dimensionless conformation tensor computed directly from the simulations corresponding to the second moment tensors of the 3-d distribution functions of R, $R^{e}$, $R^{k}$, and $R^{K}$. For the case of the end-to-end vector, this entropy turned out to be statistically indistinguishable from the exact, fully 3-d calculation according to Equation (25) under both PCF and PEF at all strain rates [25]; however, there appeared to be a small systematic error at high strain rates, but this minor drift was within the uncertainty associated with the 3-d calculation according to Equation (25).

### 4.3. Calculation of Entropy at the Atomic Scale

Entropy at the atomic scale was calculated using the method devised by Piaggi et al. [32,33] and implemented for PE melts by Nafar Sefiddashti et al. [34] to approximate the instantaneous and localized entropy projected onto each atomistic constituent. In this method, the “local” or “atomic” configurational entropy, $S_{i}$ of atom *i*, is estimated using the radial distribution (pair correlation) function according to

(59) $$S_{i}=-2\pi \rho_{N}k_{B}\int_{0}^{r_{m}}\left[g^{i}\left(r\right)\ln g^{i}\left(r\right)-g^{i}\left(r\right)+1\right]r^{2}\;dr\;\;,$$

which is the same as Equation (43) except that the upper limit of the integral has been replaced with an effective maximum radius, $r_{m}$, set equal to the cut-off radius of the RDF calculation, $r_{cut}$. In principle, this provides an instantaneous entropic snapshot of the system at each position within the simulation cell, which can be tracked as a function of time. Equation (59) was motivated by the work of Nettleton and Green [31], as discussed above, on the estimation of configurational entropy per molecule; i.e., the excess of the entropy over that of an ideal gas for low-density gases while only considering pairwise forces. As such, this expression might be subject to large absolute errors when used to estimate the entropy of dense polymeric liquids; however, here we are not interested in an accurate absolute value for the entropy of the system per se but rather in observing changes in configurational entropy arising under flow relative to the quiescent state.

To ensure that the derivatives of $g^{i}$ relative to the atomic positions were continuous, Piaggi et al. [32] used a mollified version of the RDF in Equation (59) expressed as

(60) $$g_{m}^{i}\left(r\right)=\frac{1}{4\pi \rho_{N}r^{2}}\sum_{j}\frac{1}{\sqrt{2\pi \sigma^{2}}}e^{-{\left(r-r_{ij}\right)}^{2}/\left(2\sigma^{2}\right)}\;\;,$$

where $r_{ij}$ is the distance between atoms *i* and *j* (neighbors of atom *i*), and σ is a broadening parameter. They also defined the average local entropy as

(61) $${\bar{S}}_{i}=\frac{\sum_{j}S_{j}f\left(r_{ij}\right)+S_{i}}{\sum_{j}f(r_{ij})+1}=\frac{\sum_{j}S_{j}+S_{i}}{N_{neigh}+1}$$

to increase the resolution of their method in distinguishing between different phases [33]. In this expression, $N_{neigh}$ is the number of neighbors of atom *i* within a cut-off distance $r_{c}$, and $f(r_{ij})$ is a switching function that changes from 1 at $r_{ij}\ll r_{c}$ to 0 at $r_{ij}\gg r_{c}$. Nafar Sefiddashti et al. [34] simplified the switching function to $f(r_{ij})=1$ for $r_{ij}\leq r_{c}$, and $f(r_{ij})=0$ for $r_{ij}>r_{c}$. The parameters used in Equations (59)–(61) for the results and analysis presented in this article are $r_{m}=r_{cut}=5.1$, σ=0.07, and $r_{c}=2.5$ in reduced (LJ) units. A parametric sensitivity analysis of these quantities was presented by Nafar Sefiddashti et al. [34].

A local atomic internal energy, $U_{i}$, can also be defined by adding up the contributions of the SKS potential energy in the vicinity of atom *i*. (Note that the kinetic energy contribution to the internal energy is neglected. For NVT simulations, the kinetic contribution is the same at all strain rates, and hence this term cancels out when calculating changes in internal energy relative to the quiescent state.) Similarly to $\bar{S_{i}}$, the average local internal energy can be defined as

(62) $${\bar{U}}_{i}=\frac{\sum_{j}U_{j}+U_{i}}{N_{neigh}+1}\;\;.$$

According to the above definitions of $\bar{S_{i}}$ and $\bar{U_{i}}$, which are not ensemble-averaged, these are not truly thermodynamic properties, but local thermodynamic-like quantities. Nevertheless, Legendre transformations can be applied to $U_{i}$ to obtain local versions of enthalpy, Helmholtz energy, and Gibbs energy, depending on the ensemble one prefers to use. To apply ${\bar{S}}_{i}$ and ${\bar{U}}_{i}$ in practice, the requisite parameters, $r_{cut}$, $r_{c}$, and σ, must be selected in a way that ensures the robustness of the results; as discussed by Nafar Sefiddashti et al. [34], this method is generally insensitive to the explicit choice of the model parameters and can be used without prior knowledge of the physical state of the system.

The atomistic entropy and energy as discussed above are strictly defined at the local monomer level only. According to the definitions of ${\bar{S}}_{i}$ and ${\bar{U}}_{i}$, which are not ensemble-averaged over all atoms in the system, these are not truly thermodynamic properties but local thermodynamic-like quantities that contain configurational and energetic information only within the immediate neighborhood of a particular atom. From the perspective of statistical thermodynamics, this is like examining the condition of a single subsystem of the overall thermodynamic system. Note that Equations (59)–(61) for the calculation of local entropy are implemented in LAMMPS and can be used to post-process simulation trajectories. The average ${\bar{S}}_{i}$ was calculated from the simulation output for each atom at every value of strain rate; furthermore, in several test cases, the number of atoms in the simulation cell was sufficiently large (up to 180,000 particles—see Table 1) such that instantaneous values were practically identical to the same quantities averaged over brief periods of time. To obtain an estimation of the overall state of the system, the ensemble average $\langle {\bar{S}}_{i}\rangle$ was also computed and averaged over long time durations for steady-state data. The atomic-level internal energy average $\langle U_{i}\rangle$ computed in this manner should be equivalent to 〈U〉, the time average of the total internal configurational energy of the system. (The kinetic energy contribution to the internal energy is neglected herein since only changes relative to the quiescent state were considered, and the temperature of the NVT simulations was constant at 450 K, which implies that the kinetic energy contribution is not changing with the applied strain rate). Hence, these averaged quantities resemble true thermodynamic properties of the nonequilibrium system.

## 5. Results and Discussion

### 5.1. Nonequilibrium Distribution Functions

The 3-d distribution functions required to determine entropy change (relative to the quiescent state) via Equation (25) of the C_1000_H_2002_ melt undergoing PCF and PEF were calculated from the NEMD simulations for the four segmental discretizations discussed above. Whereas these fully 3-d distributions are too complicated to display graphically, to illustrate conceptually the large variations in these distributions with respect to type of flow and flow strength, 1-d plots of the PDFs of the magnitude of the end-to-end vector, ∣R∣, as well as for the segment spanning vectors, $|R^{e}|$, $|R^{k}|$, and $|R^{K}|$ of the simulated PE melt under both steady-state PEF (left column) and PCF (right column) are displayed in Figure 3 at multiple values of the dimensionless strain rate.

It is apparent from the top-left panel of Figure 3 that the Gaussian distribution of the end-to-end vector at equilibrium remains approximately Gaussian only for very low strain rates in both types of flow. In PEF, the chains initially extend slightly with increasing strain rate (De<0.3) until a critical De (≈0.3) is attained where a coil-stretch transition occurs [51], which induces a configurational microphase separation composed of sheet-like regions of highly stretched chains enveloping ellipsoidal domains of tightly coiled macromolecules (0.3≤De≤1.5). This inhomogeneous phase manifests as bimodal PDFs [52]. At high strain rates (De≥3), the chains are almost exclusively highly stretched, providing tall, narrow distributions approaching the fully extended chain length of 1290 Å, with flow-induced crystallization eventually occurring at De≥15 as induced by random nucleation events [34,53]. Taking all into perspective, one would intuitively expect that any entropy calculations based on Gaussian statistics of a homogeneous melt would be largely inaccurate for all but the lowest De.

In PCF (top-right panel of Figure 3), the approximately Gaussian distributions of the end-to-end vector at low values of strain rate broaden significantly as Wi increases, ultimately becoming essentially bimodal at high strain rates (Wi>1000). This broadening of the PDFs is caused by the semiperiodic rotational cycles of the macromolecules induced by the vorticity of the imposed shear field after the entanglement network has begun to degrade (Wi>10) [13,44]. Under both types of flow, plots of the PDFs of the magnitude of the end-to-end vector reveal wide variations in the shape of the underlying nonequilibrium configurational distribution functions with respect to the type of flow and the flow strength that ultimately determine the entropy of the liquid. Any *a priori* notion of applying Gaussian chain statistics is eroded at all but the lowest strain rates lying within the linear viscoelastic flow regime. Once again, one would expect that entropy calculations based on the assumption of Gaussian PDFs would be highly inaccurate at all but the lowest values of Wi simulated.

The PDFs of the other segmental spanning vectors are also displayed in the various panels of Figure 3. As the contour length of the segment is reduced, even the distributions at low flow strength exhibit decreasingly Gaussian characteristics. The entanglement strand ($R^{e}$) and kink strand ($R^{k}$) vector PDFs are always unimodal, although they shift toward greater extensions with increasing strain rate and narrow (in PEF) or broaden (PCF), based on the type of applied flow. The differences between these PDFs and those of the end-to-end vector are essentially simply caused by the reduction in length of the segmental vectors, which do not allow for the high configurational freedom exhibited by R. The PDFs of the Kuhn segment vector ($R^{K}$) show relatively little change with increasing flow strength (see the bottom panels in Figure 3), especially under PCF and even at very high Wi. There is a small degree of extension of the Kuhn segments under PEF at high De, where the segments tend toward the maximum extension of about 15.6 Å when the dihedral angles are all occupying *trans* configurations.

It is common to express distribution functions in terms of their moments, such as the second-order dimensionless conformation (second moment) tensor, $C=\langle 3\;RR\rangle /R_{eq}^{2}$. This tensor then provides an ensemble-average representation of the distribution function from which it was derived. Although there is necessarily a loss of information during the averaging, generally some defining characteristics of the underlying PDFs remain. For example, the trace and determinant of the conformation tensor describe the mean-square extension of the macromolecules and directional shape of their spatial distribution. Despite the requisite loss of information, the second moment has the advantage of requiring far less statistical detail to compute than the 3-d PDFs discussed above.

**Figure 4:**
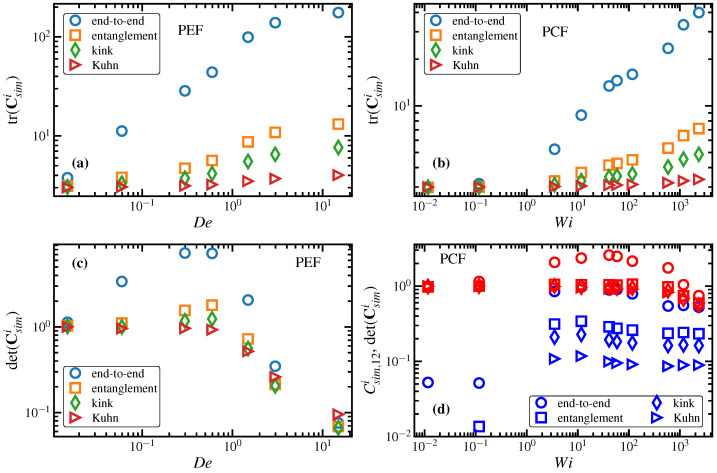
Trace and determinant of the dimensionless conformation tensor of the C_1000_H_2002_ liquid in PEF (panels (**a**,**c**)) and PCF (panels (**b**,**d**)) as a function of strain rate for all four segmental discretization levels, labelled generically as $C_{sim}^{i}$ on the ordinates. Panel (**d**), in addition to the determinant of the conformation tensor (red symbols) also depicts the $C_{sim,12}^{i}$ component in PCF with blue symbols.

The nonzero components of the symmetric conformation tensor of the end-to-end vector as determined from the simulations, $C_{sim}$, under both PCF and PEF are displayed in Figure 4 as a function of the strain rate. In both flows, trC is the sum of the diagonal components of C, each representing the mean-square extension of the macromolecules in the respective direction. In PCF, an additional independent nonzero component, $C_{sim,12}$, represents the shear-flow-induced macromolecular orientation in the flow ($x_{1}$) and the flow gradient ($x_{2}$) planes (see the blue symbols in panel (d)). The average molecular extension increases gradually at low strain rates in both PCF and PEF and then significantly at intermediate rates before reaching a plateau. As expected, the plateau value in the fractional extension reached in PEF extension is substantially greater than under PCF particularly at high deformation rates. In PCF, the $C_{sim,12}$ component increases linearly at low strain rates (see the blue symbols of panel (d): most segmental values are very close to zero (<0.01) for Wi<2 and hence not displayed in the logarithmic scale) up to Wi≈10 where the rotational cycles, i.e., macromolecular vorticity excursion, begin to appear [13,44]; hence, a maximum value is reached followed by a gradual decline as the distribution function becomes substantially broader due to the molecules becoming predominantly flow oriented on a time-averaged basis.

Figure 4 also displays plots of the nonzero components of the second moment tensors associated with other segmental discretizations for the PE melt in steady-state PEF and PCF. In all cases, the trace of the second moment tensor increases slowly at low strain rates and then increases significantly once a critical strain rate has been attained. This critical strain rate occurs at the transition to the nonlinear viscoelastic flow regime (Wi≈1) for the second moment based on the overall chain end-to-end vector (blue circles), but for other chain discretizations, this critical strain rate is pushed to higher strain rates as the size of the chain segments decreases. This is because shorter chain segments (which are energetically stiffer) actuate at shorter time scales that are only activated at higher strain rates. Note that in PCF the $C_{sim,12}^{i}$ components all exhibit a qualitatively similar behavior, each attaining a maximum as the chains orient and begin to rotate in response to the vorticity of the imposed shear field.

The determinant of the conformation tensor of the end-to-end vector initially increases in the nonlinear viscoelastic regime until it reaches a maximum at intermediate deformation rates around De≈0.5 for PEF and Wi≈40 for PCF. This increase is mostly associated with the extension of the molecules in the flow, namely, the *x* direction. At higher deformation rates, $\det\;C_{sim}$ dramatically drops as the $C_{sim,22}$ and $C_{sim,33}$ components decrease due to compression of macromolecules in the *y* and *z* directions. The segmental discretizations, $\det\;C_{sim}^{i}$, exhibit qualitatively similar behavior as $\det\;C_{sim}$; however, as expected, the ascending portion of the curves become less pronounced and they eventually merge at high strain rates as the segment sizes decrease, and in turn segments become stiffer.

### 5.2. Nonequilibrium Entropy and Free Energy

**Figure 5:**
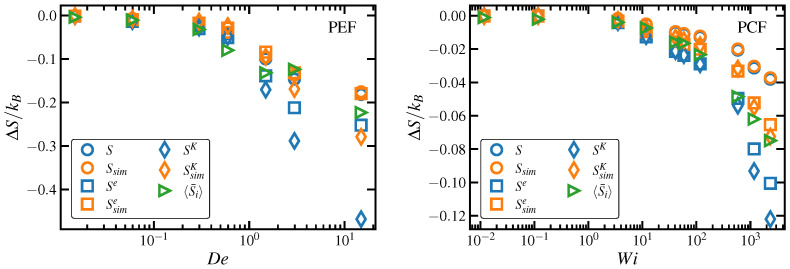
Dimensionless nonequilibrium entropy (per unit LJ volume) change (with respect to the quiescent fluid) of the C_1000_H_2002_ liquid in PEF (**left panel**) and PCF (**right panel**) as a function of strain rate. Blue symbols represent the entropy calculated from Wall’s Equation (25), using 3-d PDFs of three segmental discretization vectors (R, $R^{e}$, $R^{K}$); the orange symbols represent data calculated using the second moments according to the adaptation of Booij’s entropy expression, (58); and green symbols represent the ensemble average of the atomic entropy calculated using the approximate two-body RDF expression of Equation (59). Note that the entropy associated with the kink strands, $S^{k}$, is not displayed in the graphs for clarity since it primarily resembles that of $S^{e}$.

The entropy change, ΔS (relative to the quiescent state), as calculated from the PDFs of the end-to-end vector discussed above using Equation (25), is displayed in Figure 5 for both PCF and PEF (blue circles). The entropy decreases monotonically with increasing flow strength in both types of flow, as expected since the quiescent state represents the most probable ensemble distribution and hence the highest entropy state. The decrease is mild at low strain rates within the linear viscoelastic regime, where the individual macromolecules are still relatively coiled and their PDFs approximately Gaussian. However, at intermediate strain rates under both types of flow, the entropy drops substantially, particularly under PEF where the distributions become highly peaked at high chain extensions—see Figure 3, top-left panel—which implies a large reduction in the number of available configurations that the chain molecules can inhabit. The broad PDFs of PCF, on the other hand, result in less significant entropy changes because the wide range of available chain configurations results in higher entropy.

The entropy changes computed according to the 3-d distribution entropy equation of the segmental spanning vectors $R^{e}$ (blue squares) and $R^{K}$ (blue diamonds) under both steady-state PEF (left panel) and PCF (right panel) are also displayed in Figure 5. As the average length of the chain segments decreases, the corresponding entropy change becomes progressively larger (i.e., becomes more negative). This is because the shorter chain segments have a substantially lower number of available configurations, under both equilibrium and nonequilibrium conditions—see Figure 2 and Figure 3—and therefore the distributions become relatively broader and flatter (although over a smaller length span) as the number of chain segments is increased. As such, the difference in the number of available segment configurations relative to the equilibrium condition decreases dramatically as the number of chain segments increases, leading to progressively lower values of entropy.

It should be noted here that the statistical error associated with calculation of the entropy from Equation (25) can be quite large, and this error tends to grow as the strain rate increases and as the number of segments increases. The former trend exists because the distributions become less Gaussian with increasing strain rate (see Figure 3), which tends to make estimating the occupancy of bins at the edges of the distributions prone to larger errors. The latter trend exists because the smaller segments have less-Gaussian distributions (see Figure 2), which also exacerbates errors associated with estimating occupancies at the fringes of the distributions.

The mesoscale entropy as calculated above is theoretically exact for the choice of variable used to describe the system, within applicable statistical limitations; however, from a practical perspective, it is very computationally intensive to evaluate. First, the simulations must be highly detailed (in the present case, 180,000 atoms) and cover both very small (fs) and relatively long (ms) time scales, ultimately achieving steady-state flow conditions and maintaining these until replicable ensemble averages can be obtained. The computational requirements necessary to perform the required simulations are therefore enormous by today’s standards. Consequently, it would be very beneficial to calculate the entropy without having to introduce additional detail into the simulation, either by increasing the size of the simulation cell or increasing the run time. Unfortunately, calculation of the 3-d PDFs necessary to evaluate the entropy via Equation (25), as described above, press the extreme limits of contemporary computational resources.

Given the severe computational requirements of evaluating Equation (25) from the fully 3-d PDFs, an approximate equation for the mesoscopic entropy change could prove very valuable, provided that it could be expressed in terms of variables that were more conveniently calculated from the NEMD simulations of the C_1000_H_2002_ liquid. Such an approximate equation was presented above, (58), based on assuming that the conformation tensor of the longest relaxation mode in Booij’s entropy equation, (28), could be replaced with values of the various segmental conformation tensors computed directly from the simulation output at each value of the strain rate.

The entropy changes computed using this method, $\Delta S_{sim}^{i}$, are displayed in Figure 5 (orange symbols) as calculated from the alternative form of Booij’s entropy, Equation (58), where ΔS is the entropy change computed directly from the simulations corresponding to the second moment tensor, $C_{sim}$, of the 3-d distribution function of R, as displayed in Figure 4. As evident, this entropy is generally statistically indistinguishable from the exact, fully 3-d calculation according to Equation (25) under both PCF and PEF at all strain rates. This same trend is exhibited by the entropy as calculated using the second moment tensors of the other chain discretizations according to Equation (58), as displayed in red symbols in Figure 5. There appears to be a small systematic error at high strain rates, but this drift is within the uncertainty associated with the 3-d calculation according to Equation (25).

At high strain rates and high discretizations (i.e., shorter segments), the entropies calculated from the second moments are somewhat larger (less negative) than the corresponding entropies calculated according to 3-d distribution entropy equation. This is probably a result of the fact that using the second moments to calculate entropy provides only a rough estimation of the breadth and flatness of the fully 3-d distributions, but it could also simply be caused by statistical uncertainty associated with the calculation of the 3-d PDFs at high levels of chain discretization where the extreme edges of the distribution are very lowly populated. Another possibility, which remains to be explored, is that these discrepancies are caused by correlations between the segments as their size is reduced, rendering them nonindependent from each other, and thus effectively altering the nature of the entropy calculation increasingly as the segment size is reduced. At the level of the end-to-end vector, however, such dependency would be irrelevant since these mesostate configurations are systematically embedded in the mesostates of the more coarse-grained variable (R) chosen to describe the system. Given the statistical uncertainty associated with entropy calculations at all levels of coarse-graining, it is quite possible that all values of entropy displayed in Figure 5 at a particular strain rate are roughly equivalent, regardless of the level of coarse-graining or whether Equation (25) or Equation (58) was used to calculate them.

The implications of the preceding results are quite remarkable. Although developed for Gaussian distributions within the linear viscoelastic flow regime, from a physical perspective, Equation (58) appears to give a very good approximation of the exact system entropy, as computed using Equation (25). This is very surprising since the distributions developed in both types of flows are highly non-Gaussian (see Figure 3), and their second moments are effectively averaged over a wide range of dynamical phenomena, including flow-induced phase separation (PEF: 0.3≤De≤1.5), flow-induced crystallization (PEF: De≥15), and flow-induced rotation (PCF: Wi≥20) [13,34,44,51,52]. It is also remarkable that higher moments of the distributions are apparently not necessary to quantify the entropy, even though the distributions are highly non-Gaussian and at high flow rates that are far from the linear viscoelastic flow regime in which Equation (28) was originally derived [18,54]. The practical implication is just as remarkable since the second moments required to attain this approximation are drastically simpler to calculate than the fully 3-d PDFs required of Equation (25), and they require much less computational resources to compute. Indeed, these second moments are readily obtained, with substantial statistical certainty, directly from the NEMD simulations. Consequently, use of the approximate expression in terms of second moments greatly increases the practical utility of computing the entropy in highly nonlinear, high strain-rate flows of polymeric liquids.

The atomic entropy change based on the ensemble average $\Delta \langle {\bar{S}}_{i}\rangle =\langle {\bar{S}}_{i}\rangle -{\langle {\bar{S}}_{i}\rangle }_{eq}$ is also displayed for the C_1000_H_2002_ liquid undergoing steady-state PEF and PCF in Figure 5 as a function of the strain rate. The atomic entropy change exhibits behavior that is both qualitatively and quantitatively similar to that of the Kuhn segment entropy calculated according to the modified Booij entropy expression of Equation (58) from $C_{sim}^{K}$. In retrospect, this is possibly not surprising since atomic entropy as calculated using Equation (59) is confined to a maximum radius of $r_{m}$, which was taken as about 20 Å; this value is comparable to the Kuhn segment length of 15.6 Å. What is possibly surprising, however, is the fact that all three very different entropy Equations (25), (58) and (59), yield results that are in remarkably good agreement with each other under both types of flow and over a wide range of strain rates. The farthest outlying data points correspond to the highest strain rates of the smallest segmental vector ($R^{K}$) calculated according to Wall’s Equation (25), which are believed to possess the largest statistical uncertainty.

**Figure 6:**
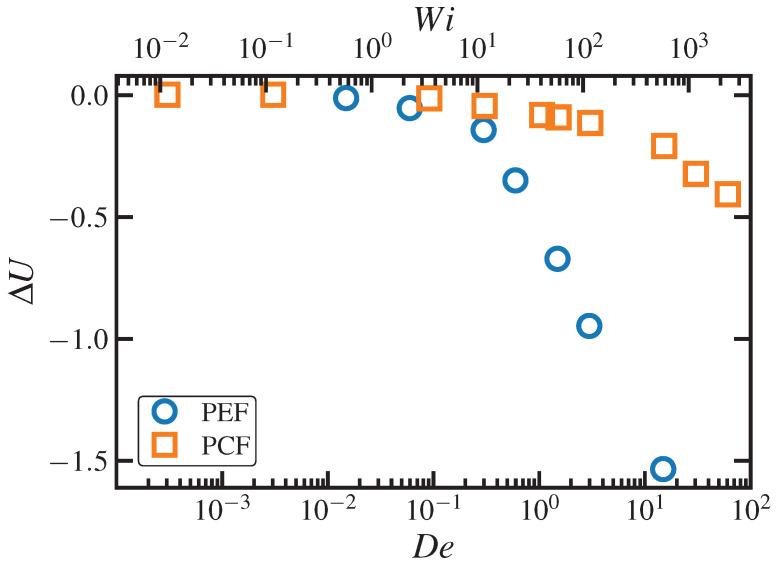
Dimensionless nonequilibrium internal energy (per unit LJ volume) change (with respect to the quiescent fluid) of the C_1000_H_2002_ liquid in PEF (blue symbols, bottom axis) and PCF (orange symbols, top axis) as a function of strain rate.

The internal energy change relative to the quiescent state of the flowing C_1000_H_2002_ liquid in both PEF (bottom axis) and PCF (top axis) is displayed in Figure 6. In both cases, the change in internal energy with the strain rate is a monotonically decreasing function, which varies only mildly in the linear viscoelastic flow regime at low strain rates. As the strain rate increases, the internal energy decline accelerates as the molecules extend under the applied flow, lowering the torsional energy of the fluid as more dihedral angles assume *trans* configurations (rather than *gauche*) and ultimately drops precipitously at high strain rates, especially in PEF where the highly stretched molecules orient and align with each other, thereby inducing a large negative change in the intermolecular LJ energy [14,53].

**Figure 7:**
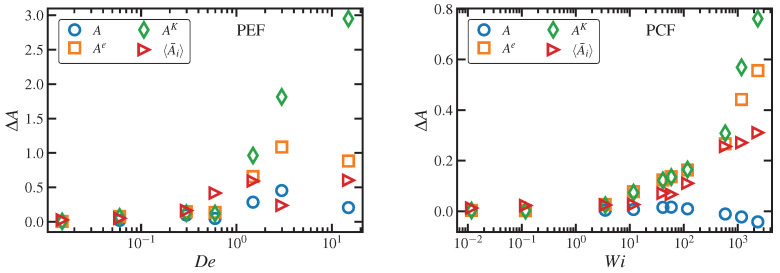
Dimensionless nonequilibrium Helmholtz-free-energy (per unit LJ volume) change (with respect to the quiescent fluid) of the C_1000_H_2002_ liquid in steady-state PEF (**left panel**) and PCF (**right panel**) as a function of strain rate. Blue, orange, and green symbols represent the Helmholtz energy calculated using the entropy from Wall’s Equation (25), using 3-d PDFs of three segmental discretization vectors (R, $R^{e}$, $R^{K}$), respectively. The red symbols represent the Helmholtz energy using the atomic entropy, $\langle {\bar{S}}_{i}\rangle$, calculated using the approximate two-body RDF expression of Equation (59).

The Helmholtz free energy change of the C_1000_H_2002_ melt under both steady-state PEF and PCF is displayed in Figure 7. This thermodynamic property, defined by the Legendre transformation ΔA=ΔU−TΔS, is rather complicated to quantify because of the delicate balance between the two opposing trends exhibited by the ΔU (monotonically decreasing) and −TΔS (monotonically increasing). In PEF, the Helmholtz energies ΔA, $\Delta A^{e}$, and $\Delta A^{K}$ all increase modestly at low De, followed by a more rapid increase at intermediate De, finally attaining a maximum and subsequently decreasing at high De (except for $\Delta A^{K}$). The behavior of the Helmholtz energy based on atomic entropy, $\langle {\bar{A}}_{i}\rangle$, is very similar, displaying a maximum at De≈8. Only $\Delta A^{K}$ displays a monotonic increase, but this might be simply a result of the rather large error associated with calculation of entropy according to Wall’s equation for such small chain segments. With the −TΔS term monotonically increasing, it is clear that the maximum and subsequent downturn in the free energy profile is caused by the decreased internal energy. Apparently, the entropy dominates the free energy at low De, whereas at intermediate De the contribution of the internal energy to the Helmholtz energy becomes a significant component of the total. At De⪆10, the molecules are sufficiently aligned and extended such that the internal energy change is large enough to overwhelm the entropic change and modify the trend of the free energy.

In PCF, a similar behavior is observed for the end-to-end vector, where the large change in internal energy at high Wi overcomes the entropy change associated with the very broad 3-d distribution functions. This behavior occurs on account of the tumbling cycles of the individual molecules, which assume many configurations over the course of a tumbling event, including some configurations that are more compressed than the random coils that exist in the quiescent liquid. As a consequence, the entropy change is not large enough to dominate the internal energy at high Wi. The behavior of the other three realizations of the Helmholtz energy, $\langle \Delta {\bar{A}}_{i}\rangle ,\;\Delta A^{e}$, and $\Delta A^{K}$, all exhibit a monotonic increase with Wi; however, each of these quantities possesses a larger entropy change than that of the end-to-end vector (see Figure 5), and consequently, the internal energy change is never of sufficient magnitude to compete with the entropic effect for these shorter chain segment variables.

What is clear from the flow behavior of the nonequilibrium free energy is that the internal energy contribution is significant and even of comparable magnitude to that of the entropy at high strain rates. In the Rouse Model of a dilute solution, the contribution of the internal energy to the free energy is zero: the macromolecular constituents have no internal energy, and there is no intermolecular energy either. In a rubber, the entanglements (i.e., cross-links) are fixed, and no significant molecular or even segmental extension can occur; hence, the behavior of these materials is almost purely entropic, as has been verified experimentally long ago. However, in a polymer melt, there is a significant reduction in the number of entanglements under flow conditions [11,12,13,22,53], and this leads to substantial molecular and segmental extension that not only changes the intramolecular internal energy but also allows for alignment of the molecules and segments that leads to substantial changes in the intermolecular internal energy. At high flow rates, where the entanglement network is effectively degraded, these internal energy changes not only become non-negligible but can also become of comparable magnitude to the entropic changes. It is likely not coincidental that the maxima in the PEF free energy curves, as well as the one based on the the end-to-end vector in PCF, roughly correspond to the strain rates where the entanglement number begins to drop precipitously in the respective flows (i.e., De≈3 in PEF [52] and Wi≈40 in PCF [13]). This contribution of internal energy to the Helmholtz free energy is a missing component in virtually all rheological theories that must be included for a successful description of the full range of flow behavior of these complicated materials.

## 6. Conclusions

Some of the challenges involved with calculating nonequilibrium entropy in flowing polymeric liquids were discussed from the perspective of internal variable theory and via atomistic NEMD simulations of a C_1000_H_2002_ polyethylene melt undergoing both steady-state planar elongational flow and planar Couette flow. The entropy change was calculated as a function of the strain rate in each type of flow at several levels of coarse-graining ranging from the end-to-end vector to the atomistic level of description. These entropies all displayed similar trends, with magnitudes that seemed to increase as the level of coarse-graining decreased but which were all of rather the same magnitude and likely within statistical error associated with the 3-d PDF calculations using Wall’s equation. It was also noted that a modified form of Booij’s entropy expression written in terms of the second moments of the PDFs approximated quite well the entropy changes computed using Wall’s equation, even though the associated distributions did not possess Gaussian characteristics. The nonequilibrium Helmholtz free energy was also calculated under both types of flow as a function of the strain rate, revealing the complicated balance between energetic and entropic effects in these macromolecular flowing liquids.

## Figures and Tables

**Table 1:** The box dimensions of planar Couette and planar elongational flow simulations at various Wi and De: $L_{x}$, $L_{y}$, and $L_{z}$ are cell lengths (in Å) in the *x*, *y*, and *z* directions, respectively, for PCF and at the initial time instant of the PEF simulations. $N_{a}$ is the number of united atoms (CH_2_ and CH_3_ units) within the cell.

Simulation	Flow Strength	$L_{x}$	$L_{y}$	$L_{z}$	$N_{a}$
PCF	0<Wi≤1.2	84.70	84.7	84.7	20,000
PCF	3.5≤Wi≤12	169.3	84.7	84.7	40,000
PCF	41≤Wi≥58	254.0	84.7	84.7	60,000
PCF	Wi≥117	508.0	84.7	84.7	120,000
PEF	0<De≤15	254.0	254	84.7	180,000

## Data Availability

All data is available on reasonable request from the authors.

## References

[1] Coleman B.D., Noll W. (1963). The thermodynamics of elastic materials with heat conduction and viscosity. Arch. Rat. Mech. Anal..

[2] Coleman B.D. (1964). Thermodynamics of materials with memory. Arch. Rat. Mech. Anal..

[3] Flory P.J. (1969). Statistical Mechanics of Chain Molecules.

[4] Leaderman H. (1941). Textile materials and the time factor. I. Mechanical behaviour of textile fibers and plastics. Text. Res..

[5] Leonov A.I. (1976). Nonequilibrium thermodynamics and rheology of viscoelastic polymer media. Rheol. Acta.

[6] Astarita G. (1974). Thermodynamics of dissipative materials with entropic elasticity. Polym. Eng. Sci..

[7] Astarita G., Sarti G.C. (1976). An approach to thermodynamics of polymer flow based on internal state variables. Polym. Eng. Sci..

[8] Beris A.N., Edwards B.J. (1994). Thermodynamics of Flowing Systems: With Internal Microstructure.

[9] Öttinger H.C. (2005). Beyond Equilibrium Thermodynamics.

[10] Dressler M., Edwards B.J., Öttinger H.C. (1999). Macroscopic thermodynamics of flowing polymeric liquids. Rheol. Acta.

[11] Nafar Sefiddashti M.H., Edwards B.J., Khomami B. (2015). Individual chain dynamics of a polyethylene melt undergoing steady shear flow. J. Rheol..

[12] Nafar Sefiddashti M.H., Edwards B.J., Khomami B. (2016). Steady shearing flow of a moderately entangled polyethylene liquid. J. Rheol..

[13] Nafar Sefiddashti M.H., Edwards B.J., Khomami B. (2019). Individual molecular dynamics of an entangled polyethylene melt undergoing steady shear flow: Steady-state and transient dynamics. Polymers.

[14] Ionescu T.C., Mavrantzas V.G., Keffer D.J., Edwards B.J. (2008). Atomistic simulation of energetic and entropic elasticity in short-chain polyethylenes. J. Rheol..

[15] Mavrantzas V.G., Theodorou D.N. (1998). Atomistic simulation of polymer melt elasticity: Calculation of the free energy of an oriented polymer melt. Macromolecules.

[16] Wall F.T. (1941). Statistical thermodynamics of rubber. J. Chem. Phys..

[17] Rouse P.E. (1953). A theory of linear viscoelastic properties of dilute solutions of coiling polymers. J. Chem. Phys..

[18] Booij H.C. (1984). The energy storage in the Rouse model in an arbitrary flow field. J. Chem. Phys..

[19] Larson R.G. (1988). Constitutive Equations for Polymer Melts and Solutions.

[20] Baig C., Edwards B.J., Keffer D.J., Cochran H.D. (2006). A comparison of simple rheological models and simulation data of n-hexadecane under shear and elongational flow. J. Rheol..

[21] Doi M., Edwards S.F. (1986). The Theory of Polymer Dynamics.

[22] Baig C., Mavrantzas V.G., Kröger M. (2010). Flow effects on melt structure and entanglement network of linear polymers: Results from a nonequilibrium molecular dynamics simulation study of a polyethylene melt in steady shear. Macromolecules.

[23] Beris A.N., Edwards B.J. (1990). Poisson bracket formulation of incompressible flow equations in continuum mechanics. J. Rheol..

[24] Beris A.N., Edwards B.J. (1990). Poisson bracket formulation of viscoelastic flow equations of differential type: A unified approach. J. Rheol..

[25] Edwards B.J., Nafar Sefiddashti M.H., Khomami B. (2021). A method for calculating the nonequilibrium entropy of a flowing polymer melt via atomistic simulation. J. Chem. Phys..

[26] Baig C., Edwards B.J. (2010). Atomistic simulation of flow-induced crystallization at constant temperature. Europhys. Lett..

[27] Baig C., Edwards B.J. (2010). Atomistic simulation of crystallization of a polyethylene melt in steady uniaxial extension. J. Non-Newton. Fluid Mech..

[28] Baig C., Edwards B.J. (2010). Analysis of the configurational temperature of polymeric liquids under shear and elongational flows using nonequilibrium molecular dynamics and Monte Carlo simulations. J. Chem. Phys..

[29] Green H.S. (1952). The Molecular Theory of Fluids.

[30] Stratonovich L.R. (1955). The entropy of systems with random number of particles. Sov. Phys. JETP.

[31] Nettleton R.E., Green M.S. (1958). Expression in terms of molecular distribution functions for the entropy density in an infinite system. J. Chem. Phys..

[32] Piaggi P.M., Valsson O., Parrinello M. (2017). Enhancing entropy and enthalpy fluctuations to drive crystallization in atomistic simulations. Phys. Rev. Lett..

[33] Piaggi P.M., Parrinello M. (2017). Entropy based fingerprint for local crystalline order. J. Chem. Phys.

[34] Nafar Sefiddashti M.H., Edwards B.J., Khomami B. (2020). A thermodynamically inspired method for quantifying phase transitions in polymeric liquids with application to flow-induced crystallization of a polyethylene melt. Macromolecules.

[35] Siepmann J.I., Karaborni S., Smit B. (1993). Simulating the critical properties of complex fluids. Nature.

[36] Mundy C.J., Siepmann J.I., Klein M.L. (1995). Calculation of the shear viscosity of decane using a reversible multiple time-step algorithm. J. Chem. Phys..

[37] Tuckerman M.E., Mundy C.J., Balasubramanian S., Klein M.L. (1997). Modified non-equilibrium molecular dynamics for fluid flows with energy conservation. J. Chem. Phys..

[38] Edwards B.J., Dressler M. (2001). A reversible problem in non-equilibrium thermodynamics: Hamiltonian evolution equations for non-equilibrium molecular dynamics simulations. J. Non-Newton. Fluid Mech..

[39] Baig C., Edwards B.J., Keffer D.J., Cochran H.D. (2005). A proper approach for nonequilibrium molecular dynamics simulations of planar elongational flow. J. Chem. Phys..

[40] Edwards B.J., Baig C., Keffer D.J. (2005). An examination of the validity of non-equilibrium molecular dynamics simulation algorithms for arbitrary steady-state flows. J. Chem. Phys..

[41] Edwards B.J., Baig C., Keffer D.J. (2006). A validation of the p-SLLOD equations of motion for homogeneous steady-state flows. J. Chem. Phys..

[42] Plimpton S. (1995). Fast parallel algorithms for short-range molecular dynamics. J. Comput. Phys..

[43] Kraynik A.M., Reinelt D.A. (1992). Extensional motions of spatially periodic lattices. Inter. J. Multiph. Flow.

[44] Nafar Sefiddashti M.H., Edwards B.J., Khomami B. (2019). Elucidating the molecular rheology of entangled polymeric fluids via comparison of atomistic simulations and model predictions. Macromolecules.

[45] Fetters L.J., Lohse D.J., Milner S.T., Graessley W.W. (1999). Packing length influence in linear polymer melts on the entanglement, critical, and reptation molecular weights. Macromolecules.

[46] Karayiannis N.C., Kröger M. (2009). Combined molecular algorithms for the generation, equilibration and topological analysis of entangled polymers: Methodology and performance. Int. J. Mol. Sci..

[47] Kröger M. (2005). Shortest multiple disconnected path for the analysis of entanglements in two-and three-dimensional polymeric systems. Comput. Phys. Comm..

[48] Hughes B.D. (1996). Random Walks and Random Environments: Vol. 1: Random Walks.

[49] Flory P.J. (1953). Principles of Polymer Chemistry.

[50] Doane D.P. (1976). Aesthetic frequency classifications. Am. Stat..

[51] Nafar Sefiddashti M.H., Edwards B.J., Khomami B. (2018). Communication: A coil-stretch transition in planar elongational flow of an entangled polymeric melt. J. Chem. Phys..

[52] Nafar Sefiddashti M.H., Edwards B.J., Khomami B. (2018). Configurational microphase separation in elongational flow of an entangled polymer liquid. Phys. Rev. Lett..

[53] Nafar Sefiddashti M.H., Edwards B.J., Khomami B. (2020). Flow-induced crystallization of a polyethylene liquid above the melting temperature and its nonequilibrium phase diagram. Phys. Rev. Res..

[54] Van Wiechen P.H., Booij H.C. (1971). A general solution to the necklace model problem in the rheology of macromolecules. J. Eng. Math..

